# Undiagnosed diabetes mellitus among older adults: a harmonized cross-country analysis of prevalence, regional disparities and risk factors in 11 European countries and Israel

**DOI:** 10.1186/s12889-025-23766-1

**Published:** 2025-08-08

**Authors:** Aijing Sun, Martina Börsch-Supan

**Affiliations:** 1 School of Management, Technical University of Munich, Munich, Germany; 2 Munich Research Institute for the Economics of Aging and SHARE Analyses, Munich, Germany

**Keywords:** Undiagnosed diabetes, Diabetes diagnosis, Risk factors, Dried blood spots (DBS), International comparisons, Public health

## Abstract

**Background:**

Undiagnosed diabetes mellitus (uDM) impacts millions of people worldwide, posing significant public health and healthcare challenges. Understanding socio-demographic, health, and healthcare factors associated with uDM is essential for reducing its prevalence, mitigating regional disparities, and enhancing early detection strategies. Since early identification of undiagnosed cases ensures timely treatment and slows the progression of complications, it saves high cost for healthcare systems.

**Methods:**

Our survey focuses on people aged 50 years and older. Based on data from the Survey of Health, Ageing, and Retirement in Europe (SHARE, Wave 6 in 2015) in 11 European countries and Israel, we combine dried blood spot (DBS) results with self-reported diabetes diagnoses to identify uDM cases. Harmonized blood spot collection and analysis across the SHARE countries enhanced the international comparability of findings. Weighted logistic regressions were applied to study factors associated with uDM compared with normoglycemia, pre- and diagnosed diabetes mellitus (dDM).

**Results:**

The prevalence of uDM in individuals aged 50 + in the SHARE countries in 2015 is 7.2%, representing 34.2% of all diabetes cases. Combined with 14.0% dDM, the total prevalence of diabetes is 21.2%. Mediterranean countries showed a higher uDM prevalence compared to northern and central European countries, ranging from 5.0% in Denmark to 14.8% in Greece. Compared to people without diabetes, undiagnosed individuals had a lower socio-economic status (SES) and were more likely to be obese and hypertensive, yet they self-rate their health similarly to those without diabetes. Compared to people diagnosed with diabetes, undiagnosed share a similar SES, but they are younger, report fewer health problems, and generally self-rate their health better.

**Conclusion:**

Early detection of diabetes is underdeveloped in Europe, with significant regional disparities. Individuals with uDM often underestimate their risks due to the asymptomatic nature of the disease in its early stages, highlighting the need for targeted public health interventions beyond the typical risk groups. Our results suggest that diabetes awareness and effective screening for uDM still need to be increased, particularly among younger and seemingly healthy individuals, those with lower SES, obesity, and hypertension.

**Supplementary Information:**

The online version contains supplementary material available at 10.1186/s12889-025-23766-1.

## Background

In its 2021 report, the International Diabetes Foundation (IDF) called diabetes mellitus “a preventable modern pandemic of unprecedented magnitude” [1]. The prevalence is growing worldwide despite decade-long efforts, and is particularly high among the older population of the high-income countries in Western Europe and the United States [2]. And while adopting sedentary lifestyles and consumption of unhealthy food, the prevalence in middle- and low-income countries is also rising and, in some regions, even overtaking Western Europe and the US [1]. In 2022, alone the United States spent over USD 421.9 billion on diabetes-related costs. In Europe, healthcare spending per person with diabetes rose from USD 2,610 in 2015 to USD 3,086 in 2021 (IDF estimate) [1, 3].

However, not all individuals living with diabetes are diagnosed or aware of their condition. Globally, undiagnosed diabetes silently impacts millions of people. Studies have shown that people with elevated blood sugar levels may remain undetected for up to 10 years [4]. Barriers to healthcare access and under-screening miss out on early asymptomatic cases, and increase the prevalence of those with pre- and undiagnosed diabetes mellitus (uDM) [5]. Without timely detection, prolonged high blood sugar levels can irreversibly affect blood vessels and organs and induce a variety of severe and costly complications, which increase the risk of cardiovascular disease (CVD), cognitive decline, chronic kidney disease (CKD), and hypertension [6–8]. In fact, initial diabetes diagnosis may come at the same time as diagnosis of diabetes complications [9].

Early diagnosis is essential not only to improve health outcomes but also to reduce long-term healthcare costs. An U.S. study found that the annual economic burden per case was USD 13,240 for diagnosed diabetes, compared to USD 4,250 for undiagnosed diabetes and even USD 500 for prediabetes [10]. Although promoting diabetes screening may initially increase healthcare spending, the long-term benefits far outweigh the expenses. A Danish study demonstrated that the modest cost of screening was offset within two years by savings in the healthcare system [11].

Accurately identifying individuals with uDM remains a challenge due to limitations in existing data sources. Information on diabetes is commonly drawn from social surveys, clinical records, or insurance claims and may have shortcomings: social surveys depend on self-reported diagnostic histories, which are susceptible to memory bias [12]; clinical records face variations in diagnostic criteria across different healthcare systems; insurance data may potentially lead to over-reporting, inflating the reported prevalence [13, 14]. Still, these data sources primarily capture information on dDM and overlook the uDM group, thereby underestimating the overall prevalence and the burden of diabetes.

An increasing number of aging-related studies [15–19] and national health surveys [20] have incorporated blood biomarker collection to provide objective health measures. However, comparing the blood data from different surveys is challenging: They use different study designs, target different age ranges, and may be conducted in different years. In addition, blood biomarkers may be analyzed from different blood specimen types e.g., venous blood (VB) vs. dried blood spots (DBS) samples in different laboratories, which will impact the biomarkers levels [18, 21, 22] complicating the comparisons or making it impossible [23]. This issue becomes particularly critical when disease or risk is defined by clinical cutoff values, as exemplified by the widely used HbA1c (glycated hemoglobin A1c) level exceeding 6.5% for diagnosing diabetes.

Here, we use harmonized data from SHARE (Survey of Health, Ageing, and Retirement in Europe) overcoming many of the aforementioned shortcomings: the blood collection strategy for all countries was harmonized, the blood samples were collected during the same time period, and analyzed in a single laboratory [18, 24]. Hence, the laboratory results are comparable across countries. In this study, we use self-reported diabetes diagnosis and HbA1c (glycated hemoglobin) blood levels measured in respondents’ DBS samples from the biomarker data set from SHARE to identify the diabetes status. We first estimate the prevalence of uDM and dDM in 11 European countries and Israel. Further, leveraging the wealth of information in the regular SHARE data set, we investigate how socio-demographic characteristics, health behaviors, and health conditions, as well as healthcare factors are associated with uDM.

Our objective is to assess the prevalence and diagnosis of diabetes among older adults in high-income European countries, address country variation, and identify which countries perform better or worse in terms of diabetes detection. In addition, we examine the characteristics that distinguish individuals with undiagnosed diabetes (uDM) from those without diabetes, as well as the factors associated with remaining undiagnosed despite having the condition. By doing so, we aim to understand which subgroups of people with diabetes are currently being overlooked and whether there are specific patterns that could help refine existing screening strategies—particularly among individuals who appear to be “seemingly healthy”. Precise estimates of diabetes prevalence and diagnosis as well as the knowledge which individuals living with diabetes remain undiagnosed are essential for assessing public health efforts, developing prevention strategies, and evaluating their effectiveness.

## Methods

### SHARE and SHARE DBS data

Although SHARE is a longitudinal panel survey, this study is based on cross-sectional data from its Wave 6 collected in 2015. Wave 6 is the only wave that included blood biomarker measurements, among them HbA1c, which is used to identify the diabetes status.

SHARE, a longitudinal nationally representative population survey launched every two years since 2004, is interviewing people aged 50 + in 27 continental European countries and Israel. In all waves, SHARE collects information on demographic and socio-economic circumstances, self-reported health, wealth and income [25].

In its Wave 6 (2015), SHARE collected blood from a finger prick as dried blood spot (DBS) samples from 27,000 respondents in 12 countries (Belgium, Switzerland, Germany, Denmark, Estonia, Spain, France, Greece, Israel, Italy, Sweden, Slovenia), in order to obtain additional objective information to complement the self-reported health status [18]. For blood biomarker assessment including HbA1c, the DBS sample was split in two randomly selected batches. Laboratory analyses for all biomarkers were performed in 2018 (Batch 1) and 2021 (Batch 2); after all, in 2023, a reassessment of HbA1c of previously analyzed samples generated the final, reliable HbA1c results [18].

In SHARE Wave 6, all eligible respondents in panel households (i.e., all who previously participated in any SHARE wave and their partners, for the latter independent of a previous participation) were asked to consent to the blood collection. People refusing, mentioning medical reasons not to donate blood, or not being able to consent by themselves were considered ineligible. Out of the total 52,374 respondents interviewed, 39,483 were eligible, of which 27,373 (67.18%) consented. In France, eligibility was restricted to only four districts (Aquitaine, Bourgogne, Bretagne, and Ile de France) due to logistical reasons. We ensured that these districts did not differ from the other French regions without DBS collection, by comparing the demographic characteristics, health conditions, and the efficiency of healthcare systems of the selected districts with those not participating and did not find significant differences (Supplementary eTable 1). Greece started blood collection only in late summer of 2015 and had the lowest DBS participation rate (33.47%) followed by Israel (52.78%). Finally, 19,611 DBS samples could be analyzed for glycosylated hemoglobin (HbA1c). For the selection of the study population and a detailed description of the collection, processing, and analysis of the SHARE DBS samples see Börsch-Supan, M et al., 2024 [18].

### The HbA1c and diabetes group classification

Glycated hemoglobin (HbA1c) reflects blood glucose levels over the past two to three months. It is commonly used as a blood glucose test in individuals with diabetes, but also in population screening for diabetes as fasting is not necessary [26]. According to the clinical guidelines of the American Diabetes Association (2013), HbA1c values below 5.7% indicate normoglycemia, while values between 5.7% and 6.4% are classified as prediabetes. HbA1c values ≥ 6.5% are diagnostic of diabetes [27].

**Fig. 1:**
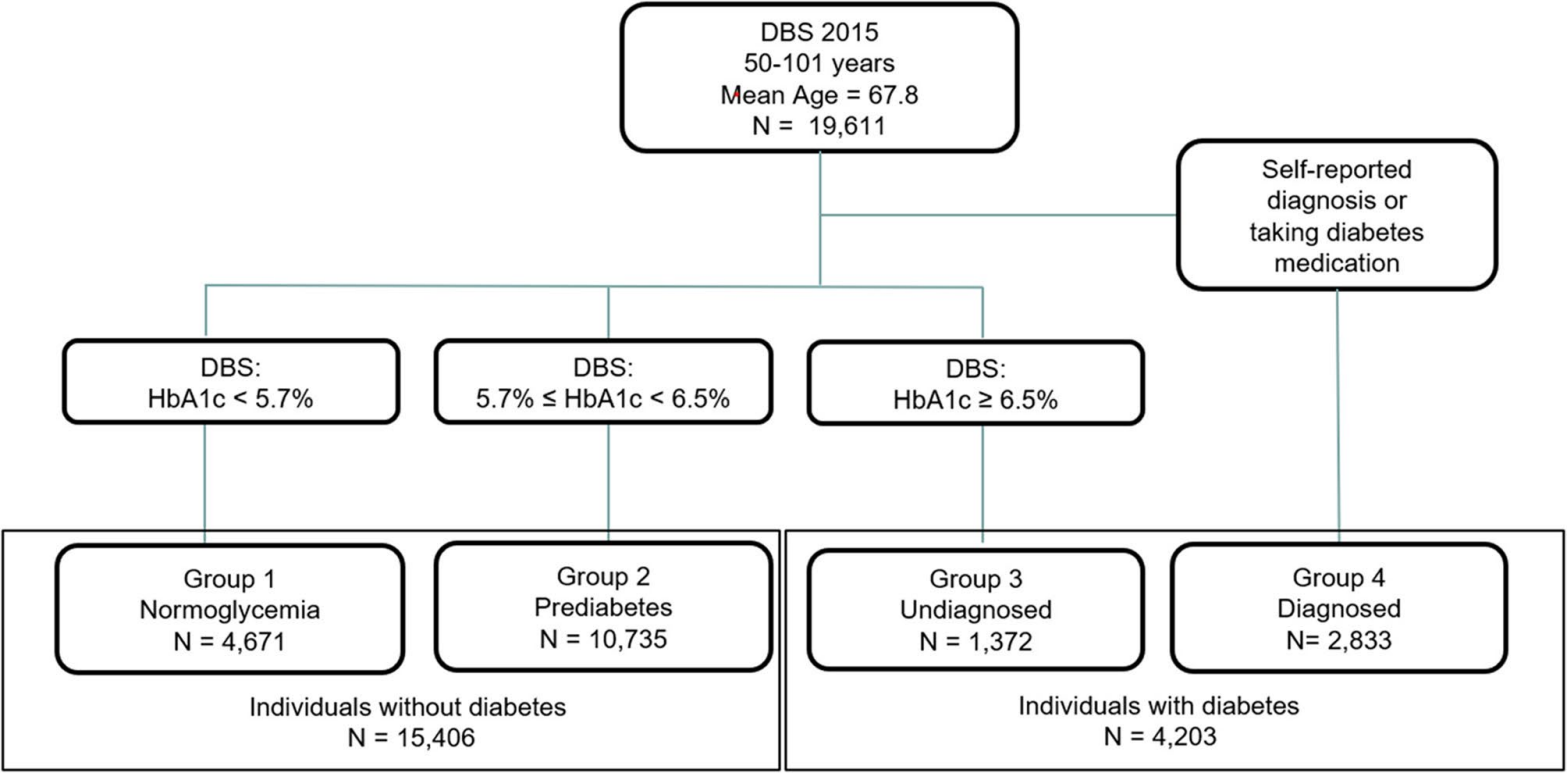
Sample selection and diabetes group classification flowchart in SHARE sample Source: The Survey of Health, Ageing and Retirement in Europe, Wave 6 in 2015, 11 European countries and Israel

Our study groups DBS participants into four categories by the SHARE-measured HbA1c level and self-reported diabetes diagnosis. Since medication can lower HbA1c levels, we classify people who report either being diagnosed by a doctor or taking diabetes medication as diagnosed (dDM; group 4). This includes 130 individuals who deny a diabetes diagnosis by a doctor but report taking diabetes medication. Those neither diagnosed by a doctor nor taking medication fall into three groups according to their measured HbA1c level: Group 1 (normoglycemia, HbA1c < 5.7%), group 2 (preDM, HbA1c between 5.7 and 6.5%), and group 3 (uDM, HbA1c ≥ 6.5%); the latter includes individuals never diagnosed by a doctor nor taking related medications, but having unknowingly a blood-glucose level that meets the definition of diabetes. Individuals in groups 1 and 2 are considered as individuals without diabetes, whereas those in group 3 (uDM) and group 4 (dDM), are classified as individuals with diabetes. Figure 1 shows the flowchart for the classification of the diabetes groups.

### Covariates

To investigate the factors potentially associated with uDM, we reviewed relevant literature from population as well as clinical studies. We hypothesize that individuals with undiagnosed diabetes may have different socio-demographic background, health behaviors, health conditions, and a different utilization of healthcare services compared to individuals without diabetes or those diagnosed with the condition.

#### Demographic factors

We controlled for age, gender, and immigration background as well as cohabitation status. We created four age groups by decade: 50–60, 61–70, 71–80, and 80+. Immigration background: we distinguished if individuals were born in the respective SHARE country or elsewhere. Cohabitation status: being married and living with a partner indicates not living alone; living separately from a spouse, never having been married, being divorced or widowed was categorized as living alone.

#### Socio-economics status

We control for individual’s education and household income. Education systems vary widely between countries in terms of structure, duration, and curriculum. Instead of using years of education, we group people into Low (pre-primary or lower secondary), Medium (upper secondary or post-secondary non-tertiary), and High (tertiary, including undergraduate and postgraduate degrees) education based on ISCED - International Standard Classification of Education. Considering the economic differences between countries, household income is categorized into four quantiles reflecting its distribution relative to national income levels.

#### Health behaviors

Health behaviors associated with an increased risk of diabetes are incorporated into our analysis. Smoking status is classified whether individuals have ever smoked. Physical inactivity is defined as rarely or never engaging in moderate or vigorous exercise. Alcohol consumption is assessed through the question: “In the last three months, how often did you consume six or more units of alcoholic beverages on one occasion?” (Reporting “Not at all” = non-drinkers, “, once or twice a month” = occasional drinkers, and “more than once a week” = frequent drinkers).

#### Health conditions

We include both self-assessed health status and respondent-reported diagnosed conditions from SHARE Wave 6 in our analysis. Individuals are categorized into three groups based on their self-assessed health status: “Excellent/very good “, “Good”, and “Fair/Poor”. Further, we include the following self-reported diagnoses: hypertension, high blood cholesterol, stroke, chronic kidney disease, and mental health conditions such as Alzheimer’s disease, dementia, and senility. Adiposity is assessed from BMI (Body Mass Index): normal (BMI ≤ 25 kg/m²), overweight (BMI 25 to 29.9 kg/m²), obese (BMI ≥ 30 kg/m²). Currently, the relationship between depression and diabetes is receiving significant attention. Therefore, our analysis includes a binary variable to determine if individuals have experienced depression.

#### Healthcare usage

We aim to explore whether individuals with undiagnosed diabetes use healthcare services differently and/or face greater barriers in healthcare access. We assess healthcare utilization with a binary variable to identify whether individuals have visited a doctor within the past 12 months. Additionally, we examine barriers to healthcare by using two binary variables indicating whether individuals have forgone doctor visits due to long waiting times or high costs, respectively.

### Statistical methods

The purpose of this study is to estimate the prevalence and diagnosis of uDM at the national population level. To address potential selection bias resulting from the SHARE survey participants and the process of blood sample collection, we apply DBS-specific weights. The weights consist of two components: the first component corrects the bias between the national population and non-response to the entire SHARE survey participation; the second component additionally adjusts for non-response in the blood-sampling module among SHARE Wave 6 participants, accounting for biases in gender, age, and education. The derivation of DBS weights is described in Börsch-Supan, M. et al., 2024 [18].

The diabetes prevalence is estimated using a weighted logistic regression. To facilitate fair cross-country comparisons and remove differences attributable to socio-demographic structure, both unadjusted and adjusted prevalence rates were calculated. The unadjusted rates reflect the actual age, gender, and education distribution within each country, while adjusted prevalence was estimated by standardizing for age, gender, and education distribution to match the average across these countries. Specifically, we estimated a logistic regression model predicting diabetes prevalence, keeping the distribution of age, gender, and education in the average values across the 12 countries. This approach allows us to isolate differences in diabetes prevalence that are not due to population structure, ensuring comparability across countries. To explore regional differences in uDM prevalence, we classify countries into three European regions: northern (Sweden, Estonia, Denmark), central (Belgium, Germany, Switzerland, France, Slovenia), Mediterranean countries (Italy, Spain, Greece), and Israel.

Next, we conducted a series of weighted logistic regressions to understand the factors associated with uDM compared to individual with normoglycemia, prediabetes, and diagnosed diabetes, respectively. Odds ratios (ORs) were reported for each regression. Our analysis is performed in two stages: (1) controlling for socio-demographic characteristics, (2) adding controls for health behaviors, health conditions, and use of healthcare. In the regression comparing uDM to dDM, we did not adjust for healthcare utilization, as individuals with diagnosed diabetes are more likely to visit healthcare providers regularly, resulting in greater exposure to the healthcare system. This could introduce the issue of reverse causality. Since BMI is widely recognized as a key risk factor for diabetes onset and progression, we conducted additional subgroup analyses by BMI category (normal weight, overweight, and obese) to isolate the associations between other factors and uDM independently of BMI variation.

All models incorporate country-fixed effects, using Sweden as reference, to account for variations between countries not explained by the controlled factors. A total of 478 out of 19,611 observations had missing values in some control variables, as this number is relatively small compared to the overall sample size, these observations were omitted from the regression analysis through listwise deletion. We considered p-values below 5% as significant. All statistical analyses are conducted using STATA, version 14.

### Sensitivity analysis

Diabetes is diagnosed when the HbA1c level is ≥ 6.5%. We follow this threshold definition in our analysis, which may potentially lead to misclassifications due to factors such as analytical variability in testing conditions. However, our measure for HbA1c has been carefully corrected for fieldwork conditions [18]. To further ensure the robustness of our findings, we conducted a sensitivity analysis using a stricter threshold for diabetes diagnosis of 7%. Specifically, among individuals not classified as dDM, we reclassified those with HbA1c ≥ 7.0% as uDM, and those with HbA1c < 7.0% as individuals without diabetics and then repeated our analysis.

## Results

A total of 19,611 observations were included in the study, covering individuals aged 50 to 101 years, with a mean age of 67.8 years. Based on our diabetes classification, 4,671 individuals were normoglycemic, 10,735 had reached the prediabetic state, 1,372 had undiagnosed diabetes, and 2,833 had diagnosed diabetes as shown for each group in Fig. 1.

### The prevalence of diabetes by age and gender

**Fig. 2:**
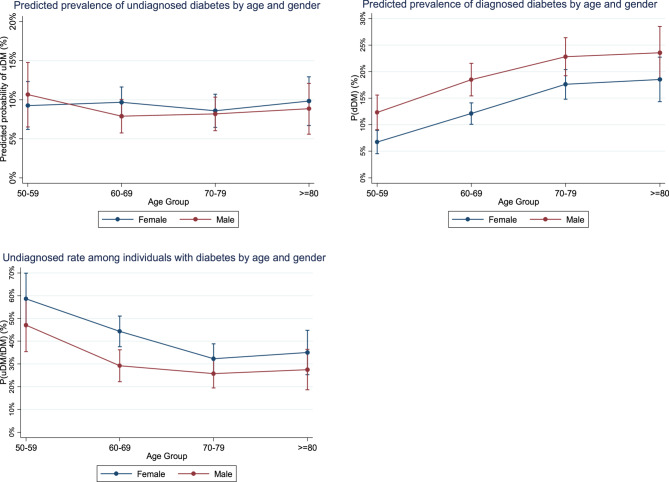
The prevalence of undiagnosed and diagnosed diabetes, along with the undiagnosed rate among individuals with diabetes (uDM/tDM) by age and gender groups Source: The Survey of Health, Ageing, and Retirement in Europe, Wave 6 in 2015, 11 European countries and Israel Notes: The prevalence estimates are weighted to the population distribution. The x-axis represents age groups (50–59, 60–69, 70–79, and ≥80 years). uDM = undiagnosed diabetes mellitus; dDM = diagnosed diabetes mellitus; tDM = total diabetes mellitus (diagnosed + undiagnosed); uDM/tDM = proportion of undiagnosed diabetes among all diabetes cases. Error bars represent 95% confidence intervals

Figure 2 shows the prevalence patterns of uDM compared to dDM across age and gender groups. While the uDM prevalence remains relatively stable across age groups and gender, dDM prevalence exhibits an upward trend from ages 50 to 79 and stabilizes after age 80, with males showing a higher prevalence than females. However, within the population with diabetes (tDM), the proportion of undiagnosed (uDM/tDM) decreases with age, and is significantly higher in females compared to males. Detailed estimations of prevalence rates across age and gender groups are presented in Supplementary eTable 2

### The prevalence of undiagnosed diabetes across countries and its regional disparities

The prevalence of uDM among individuals aged 50 + across 12 SHARE countries was 7.2% [5.8–9.0] in 2015. Combined with 14.0% [12.1–16.0] prevalence of dDM, the total diabetes prevalence (tDM) reached 21.2% [19.0–23.6]. Notably, among all individuals with diabetes, 34.2% [28.6–40.2] of them were undiagnosed. The overall prevalence across all 12 countries, as well as the adjusted prevalence rates for each individual country, are reported in Table 1. Unadjusted prevalence estimates are provided in Supplementary eTable 3. After adjustment, the prevalence rates changed only slightly. We also include the prevalence of prediabetes in supplementary eTable 3, and find that approximately half of the population has prediabetic A1c blood levels.

**Table 1:** Adjusted prevalence of undiagnosed and diagnosed diabetes, and the undiagnosed rate (uDM/tDM) in adults aged 50 and older

Country	uDM (%)	dDM (%)	tDM (%)	uDM/tDM (%)
Across countries	7.2	14.0	21.2	34.2
	[5.8–9.0]	[12.1–16.0]	[19.0–23.6]	[28.6–40.2]
By Country				
Northern EU				
Sweden	7.0	11.9	18.9	36.4
	[7.0–7.0]	[11.9–11.9]	[16.7–21.1]	[36.3–36.5]
Estonia	5.1	15.8	20.9	23.8
	[5.1–5.2]	[15.7–15.9]	[19.3–22.6]	[23.5–24.1]
Denmark	5.0	9.0	14.0	36.2
	[5.0–5.1]	[9.0–9.0]	[12.4–15.6]	[36.0–36.4]
Central EU				
Belgium	6.0	13.2	19.2	31.3
	[5.9–6.0]	[13.2–13.2]	[17.3–21.0]	[31.2–31.4]
Germany	8.5	17.4	26.0	32.5
	[8.5–8.6]	[17.4–17.4]	[23.5–28.4]	[32.4–32.5]
Switzerland	5.4	8.3	13.6	41.6
	[5.4–5.4]	[8.2–8.3]	[11.8–15.5]	[41.4–41.8]
France	8.3	10.3	18.6	47.9
	[8.3–8.3]	[10.3–10.3]	[13.7–23.5]	[47.8–47.9]
Slovenia	9.6	16.3	25.8	36.8
	[9.5–9.6]	[16.2–16.4]	[23.1–28.6]	[36.6–37.0]
Mediterranean				
Italy	9.3	13.5	22.8	40.7
	[9.3–9.3]	[13.5–13.5]	[19.9–25.7]	[40.7–40.8]
Spain	13.4	19.6	33.0	38.4
	[13.4–13.4]	[19.6–19.6]	[28.0–38.0]	[38.4–38.5]
Greece	14.8	18.8	33.6	44.2
	[14.8–14.8]	[18.7–18.8]	[29.1–38.1]	[44.1–44.3]
Israel	10.1	32.9	43.0	22.4
	[10.1–10.2]	[32.8–33.0]	[34.6–51.5]	[22.3–22.6]

Source: The Survey of Health, Ageing, and Retirement in Europe, Wave 6 in 2015, 11 European countries and Israel

Note: *uDM* Undiagnosed diabetes mellitus, *dDM* Diagnosed diabetes mellitus, *tDM* Total diabetes mellitus (combined uDM and dDM). The uDM/tDM ratio indicates the proportion of undiagnosed cases among all individuals with diabetes. Adjusted prevalence rates were estimated by standardizing for age, gender, and education to the average distribution across countries, holding these factors constant. All estimates are weighted to reflect population distribution. Values in brackets represent 95% confidence intervals

**Fig. 3:**
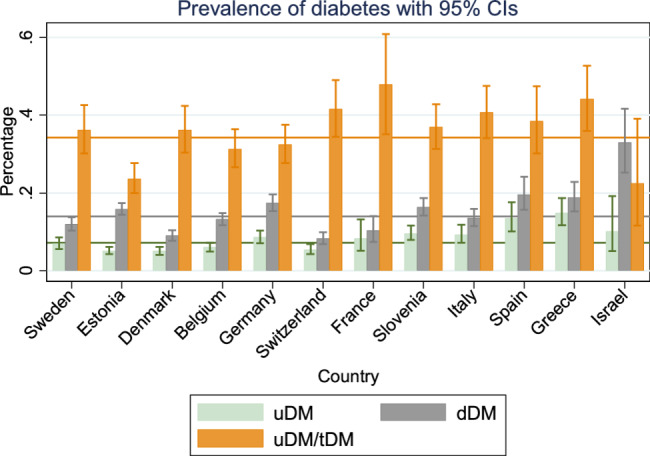
Regional variations in adjusted prevalence of diagnosed and undiagnosed diabetes, and undiagnosed rate among (uDM/tDM) Source: The Survey of Health, Ageing, and Retirement in Europe, Wave 6 in 2015, 11 European countries and Israel Note: The prevalence rates were adjusted by standardizing for age, gender, and education levels to the average levels observed across multiple countries, thereby holding these factors constant across different populations. Green bars indicate uDM (undiagnosed diabetes mellitus), the green horizontal line is the average prevalence of uDM (7.2%) across countries. Gray bars represent dDM, the gray horizontal line is the average prevalence of dDM (14.0%) across countries. Orange bars represent the undiagnosed rate among all individuals with diabetes, with the orange horizontal line representing the average undiagnosed rate (34.2%) across countries

Significant disparities in uDM prevalence are observed between the countries (Fig. 3): northern and central European countries have a lower uDM prevalence than Mediterranean countries. Specifically, Sweden, Denmark, Belgium, and Switzerland have an estimated uDM prevalence below the average across all countries. In the Mediterranean countries Italy, Israel, Spain, and Greece, uDM prevalence is higher. These disparities are also mirrored in dDM prevalence. Consequently, when considering both uDM and dDM, the tDM prevalence highlights a pronounced north-south gradient. Specifically, Denmark and Switzerland have the lowest tDM prevalence at around 14%, while in Spain and Greece the overall diabetes prevalence is approximately 33%. Israel records the highest prevalence in the older population among the SHARE countries at 43.6%. These findings underscore that approximately one in three individuals aged 50 + in Spain and Greece and almost every second in Israel have diabetes.

Among the population with diabetes, the proportion of undiagnosed diabetes (uDM/tDM) also shows regional disparities, with Mediterranean countries and parts of continental Europe having higher uDM proportions compared to northern Europe: more than 40% of diabetes cases remain undiagnosed in Switzerland (41.6%), France (47.9%), Greece (44.2%), and Italy (40.7%). Switzerland and France—despite the relatively low total diabetes prevalence (13.6% and 18.6%, respectively)—exhibit high undiagnosed proportions, whereas Italy and Greece show both high total diabetes prevalence (22.8% and 33.6%, respectively) and high shares of undiagnosed cases. In contrast, Estonia has the lowest undiagnosed rate (23.8%), and Israel also shows a similarly low rate of 22.4%, but due to a small Israeli sample size, the estimate has a wide confidence interval.

### Factors associated with undiagnosed diabetes

Table 2 presents the results from a serious of weighted logistic regression models assessing the risk factors associated with undiagnosed diabetes (uDM), in comparison with three different groups: normoglycemic individuals (Columns 1–2), individuals with prediabetes (Columns 3–4) and individual with diagnosed diabetes (Columns 5–6). Models (1), (3), and (5) include only socio-demographic and socio-economic variables, while Models (2), (4), and (6) additionally control for health behaviors, health conditions, and healthcare use. A total of 478 observations with missing values in control variables were excluded, resulting in a final analytical sample of 19,133 individuals. Descriptive statistics for the study population stratified by country are provided in Supplementary eTable 4, and by diabetes classification group in eTable 5.

Compared to normoglycemic individuals, people with uDM are significantly more likely to be obese (OR = 2.01, 95% CI [1.30–3.12]), and to have hypertension (OR = 1.80, 95% CI [1.27–2.55]) (Model 2). Higher educational attainment is associated with lower odds of having uDM, although the effect attenuates after controlling for health conditions and behaviors, suggesting that the effect of education on diabetes risk may be mediated through these intermediate factors. There are no significant differences in the demographic distribution, i.e., age, gender, cohabitation, or immigration background, between the undiagnosed and normoglycemic individuals. Furthermore, regional disparities persist after controlling for all observed characteristics: compared to Sweden, individuals from Denmark have lower odds of uDM (OR = 0.51), while those from some central European and Mediterranean countries like Slovenia (OR = 1.60), Spain (OR = 2.74), and Greece (OR = 4.149, *p* < 0.01) have significantly higher odds of uDM.

When compared to individuals with prediabetes, those with uDM still show a strong association with hypertension (OR = 1.52, 95% CI [1.14–2.04]). High education remains associated with lower odds of uDM (OR = 0.61, 95% CI [0.40–0.94]), and this effect persists after controlling for other variables. However, differences in BMI and many other health-related behaviors are no longer significant. Nevertheless, clear regional variation persists: odds of uDM remain significantly higher in Israel (OR = 3.82), Greece (OR = 2.66), and Spain (OR = 2.45), while significant lower in Estonia (OR = 0.65) compared to Sweden.

Compared to individuals with diagnosed diabetes, we observe that females, younger individuals, and individuals born in the country had higher odds of remaining undiagnosed at the time of blood sample collection. No significant association is evident concerning socio-economic status, i.e., education level and household income level. Individuals with well-known diabetes risk factors or comorbidities are associated with lower odds of being undiagnosed, suggesting those comorbidities are more common among individuals who have already received a diagnosis. Specifically, individuals with overweight (OR = 0.57, 95% CI [0.40–0.82]), obesity (OR = 0.32, 95% CI [0.21–0.49]), physical inactivity (OR = 0.59, 95% CI [0.38–0.93]), or high blood cholesterol (OR = 0.42, 95% CI [0.30–0.61]) are more likely to have been diagnosed. Compared to Sweden, only France shows a significantly higher rate of undiagnosed diabetes (OR = 2.70, 95% CI [1.30–5.63]).

**Table 2:** Factors associated with undiagnosed diabetes mellitus (uDM), with reported odds ratios and 95% confidence intervals

	**Variables**	(1)	(2)	(3)	(4)	(5)	(6)
		Undiagnosed vs Normoglycemic	Undiagnosed vs Normoglycemic	Undiagnosed vs Prediabetic	Undiagnosed vs Prediabetic	Undiagnosed vs Diagnosed	Undiagnosed vs Diagnosed
*Social Demographics*	Male (ref: Female)	1.07	1.03	1.13	1.14	0.59***	0.57***
		[0.77 - 1.48]	[0.73 - 1.45]	[0.86 - 1.50]	[0.84 - 1.54]	[0.44 - 0.78]	[0.41 - 0.77]
	Age 60–69 (ref: 50–59)	1.07	0.95	0.81	0.76	0.49***	0.54***
		[0.74 - 1.56]	[0.66 - 1.38]	[0.58 - 1.15]	[0.53 - 1.08]	[0.33 - 0.74]	[0.36 - 0.82]
	Age 70-79	1.19	0.98	0.73*	0.66**	0.36***	0.45***
		[0.79 - 1.78]	[0.64 - 1.50]	[0.51 - 1.05]	[0.45 - 0.97]	[0.23 - 0.54]	[0.29 - 0.67]
	Age 80+	1.31	1.15	0.77	0.67	0.44***	0.55**
		[0.77 - 2.22]	[0.68 - 1.97]	[0.48 - 1.23]	[0.41 - 1.10]	[0.27 - 0.71]	[0.33 - 0.91]
	Immigrant (ref: born native)	0.79	0.81	0.87	0.88	0.50**	0.52**
		[0.44 - 1.42]	[0.46 - 1.43]	[0.52 - 1.46]	[0.54 - 1.43]	[0.28 - 0.88]	[0.29 - 0.95]
	Cohabiting (ref: non-cohabiting)	1.09	1.15	0.90	0.89	1.08	1.13
		[0.78 - 1.53]	[0.82 - 1.61]	[0.66 - 1.22]	[0.66 - 1.21]	[0.77 - 1.54]	[0.78 - 1.63]
*Socio-economic Status*	Medium Edu (ref: Low Edu)	0.95	1.01	0.87	0.92	1.04	0.99
		[0.60 - 1.50]	[0.64 - 1.58]	[0.60 - 1.28]	[0.63 - 1.32]	[0.70 - 1.54]	[0.67 - 1.47]
	High Edu	0.56**	0.63*	0.60**	0.61**	1.24	0.96
		[0.35 - 0.90]	[0.38 - 1.03]	[0.39 - 0.92]	[0.40 - 0.94]	[0.80 - 1.91]	[0.62 - 1.48]
	Income 2nd quantiles (ref: 1st)	1.03	1.05	0.94	0.95	1.18	0.99
		[0.64 - 1.64]	[0.66 - 1.67]	[0.62 - 1.42]	[0.64 - 1.40]	[0.79 - 1.76]	[0.66 - 1.49]
	Income 3rd quantiles	0.96	0.98	1.06	1.06	1.51*	1.15
		[0.59 - 1.55]	[0.63 - 1.54]	[0.71 - 1.59]	[0.71 - 1.59]	[0.97 - 2.34]	[0.72 - 1.85]
	Income 4th quantiles	0.65	0.66	0.81	0.83	1.39	1.10
		[0.38 - 1.13]	[0.39 - 1.10]	[0.50 - 1.31]	[0.52 - 1.33]	[0.84 - 2.30]	[0.65 - 1.87]
*Health Behavior*	Physical Inactivity		1.00		0.93		0.59**
			[0.59 - 1.69]		[0.61 - 1.41]		[0.38 - 0.93]
	Ever Smoked		1.09		0.98		0.91
			[0.80 - 1.49]		[0.73 - 1.31]		[0.67 - 1.22]
	Alcohol Use: Moderate (ref: No)		0.82		0.85		1.39
			[0.48 - 1.43]		[0.50 - 1.45]		[0.77 - 2.52]
	Alcohol Use: Frequent		0.62*		0.86		1.50
			[0.35 - 1.07]		[0.53 - 1.39]		[0.73 - 3.11]
*Health Condition*	Self-rated Health: Good (ref: Very good/excellent)		0.93		0.82		0.41***
			[0.64 - 1.34]		[0.59 - 1.14]		[0.25 - 0.67]
	Self-rated Health: Fair/Poor		1.03		1.10		0.25***
			[0.65 - 1.63]		[0.74 - 1.62]		[0.14 - 0.44]
	BMI 25–29.9 (Overweight, ref: <25 Normal)		1.39*		1.01		0.57***
			[1.00 - 1.93]		[0.75 - 1.35]		[0.40 - 0.82]
	BMI ≥30 (Obese)		2.01***		1.01		0.32***
			[1.30 - 3.12]		[0.71 - 1.45]		[0.21 - 0.49]
	Hypertension		1.80***		1.52***		0.89
			[1.27 - 2.55]		[1.14 - 2.04]		[0.65 - 1.22]
	High Blood Cholesterol		0.92		0.73*		0.42***
			[0.63 - 1.33]		[0.53 - 1.00]		[0.30 - 0.61]
	Stroke		1.62		1.11		0.76
			[0.68 - 3.90]		[0.50 - 2.47]		[0.38 - 1.52]
*Health Condition*	Mental Health		0.95		0.55		0.65
			[0.36 - 2.49]		[0.21 - 1.50]		[0.25 - 1.68]
	Chronic Kidney Disease		1.19		0.91		0.84
			[0.42 - 3.40]		[0.39 - 2.10]		[0.36 - 1.98]
	Depression		0.95		0.97		1.31
			[0.68 - 1.31]		[0.74 - 1.27]		[0.94 - 1.81]
	Sleep Problem		1.00		0.88		0.91
			[0.66 - 1.50]		[0.64 - 1.20]		[0.64 - 1.29]
*Healthcare Usage*	Doctor Visit Last Year		1.04		0.92		
			[0.64 - 1.68]		[0.60 - 1.40]		
	Forgo care - cost		1.12		0.75		
			[0.50 - 2.53]		[0.41 - 1.40]		
	Forgo care - long wait		0.85		1.21		
			[0.44 - 1.66]		[0.65 - 2.24]		
Country	Estonia (baseline: Sweden)	0.82	0.74	0.74**	0.65**	0.58***	1.01
		[0.59 - 1.15]	[0.50 - 1.09]	[0.54 - 1.00]	[0.46 - 0.93]	[0.40 - 0.84]	[0.65 - 1.57]
	Denmark	0.49***	0.51***	0.78	0.81	0.98	0.82
		[0.35 - 0.68]	[0.36 - 0.72]	[0.57 - 1.06]	[0.58 - 1.13]	[0.67 - 1.45]	[0.52 - 1.30]
	Belgium	0.76	0.81	0.88	0.98	0.72*	0.81
		[0.54 - 1.06]	[0.57 - 1.16]	[0.65 - 1.20]	[0.71 - 1.36]	[0.50 - 1.05]	[0.52 - 1.27]
	Germany	1.36*	1.35*	1.34*	1.35*	0.87	1.10
		[0.97 - 1.90]	[0.96 - 1.92]	[0.99 - 1.82]	[0.97 - 1.87]	[0.61 - 1.25]	[0.72 - 1.69]
	Switzerland	0.70*	0.74	0.76	0.79	1.31	1.18
		[0.48 - 1.02]	[0.51 - 1.09]	[0.54 - 1.07]	[0.56 - 1.13]	[0.85 - 2.01]	[0.73 - 1.92]
	France	0.95	0.99	1.42	1.53	1.66	2.70***
		[0.51 - 1.75]	[0.55 - 1.76]	[0.79 - 2.55]	[0.87 - 2.66]	[0.87 - 3.15]	[1.30 - 5.63]
	Slovenia	1.76***	1.60**	1.39**	1.41*	1.02	1.50
		[1.22 - 2.56]	[1.08 - 2.38]	[1.01 - 1.93]	[0.99 - 1.99]	[0.69 - 1.52]	[0.92 - 2.44]
	Italy	1.28	1.31	1.48**	1.49*	1.08	1.45
		[0.85 - 1.94]	[0.83 - 2.07]	[1.02 - 2.16]	[1.00 - 2.21]	[0.71 - 1.66]	[0.88 - 2.39]
	Spain	2.58***	2.74***	2.29***	2.45***	0.91	1.15
		[1.52 - 4.38]	[1.59 - 4.72]	[1.52 - 3.45]	[1.59 - 3.78]	[0.56 - 1.49]	[0.69 - 1.93]
	Greece	3.92***	4.14***	2.42***	2.66***	1.26	1.72*
		[2.45 - 6.28]	[2.49 - 6.88]	[1.64 - 3.57]	[1.75 - 4.04]	[0.78 - 2.04]	[0.99 - 3.01]
	Israel	1.24	1.19	3.77***	3.82***	0.82	1.07
		[0.52 - 2.94]	[0.50 - 2.82]	[1.71 - 8.32]	[1.76 - 8.28]	[0.36 - 1.89]	[0.47 - 2.46]
	Observations	5,896	5,896	11,848	11,848	4,065	4,065

Source: The Survey of Health, Ageing, and Retirement in Europe, Wave 6 in 2015, 11 European countries and Israel

Note: Table presents odds ratios (ORs) and 95% confidence intervals [in brackets] from weighted logistic regression models. All models include country fixed effects (Sweden as reference) and are weighted to reflect population distribution. Models compare individuals with undiagnosed diabetes (uDM) to normoglycemic (Cols 1–2), prediabetes (Cols 3–4), and diagnosed diabetes (Cols 5–6). Models in Cols 1, 3, and 5 adjust for sociodemographic; Cols 2, 4, and 6 additionally control for health behaviors, conditions, and healthcare use

Statistical significance: *p < 0.1, **p < 0.05, and ***p < 0.01

Subgroup analyses by BMI category (normal, overweight, obese) revealed broadly consistent patterns with key variations (Supplementary eTables 6–8). When comparing uDM to normoglycemia (eTables 6), among individuals with normal BMI, higher education was significantly associated with lower odds of undiagnosed diabetes (uDM), but this protective effect diminished in overweight and obese groups, where health-related risk factors - such as stroke, hypertension, smoking, high cholesterol, and depression - became stronger correlates of uDM. When comparing uDM to prediabetes (eTables 7), no significant differences were found in the normal BMI group, while in higher BMI groups, uDM was rather linked to hypertension, and prediabetes to stroke, kidney disease, and depression. Compared to diagnosed diabetes (eTable 8), uDM remained more likely among those with healthier lifestyles across all BMI groups, though alcohol use was an exception. Overall, the protective effect of education was most pronounced among individuals with lower metabolic risk.

### Sensitivity analysis

After applying a stricter diagnostic threshold by increasing the HbA1c cutoff from 6.5 to 7%, individuals with mildly elevated blood glucose levels (HbA1c between 6.5% and 7%) are reclassified from the uDM to the preDM group. Consequently, the sample size of the uDM group decreases from 1,338 to 404 with 934 individuals (69%) being reclassified as preDM (Supplementary eTable 9). Despite this shift, most key associations remained consistent with the main results (Supplementary eTable 10 vs. Table 2).

Under the stricter definition, associations with overweight and obesity became stronger when comparing uDM to normoglycemic and prediabetic individuals, reinforcing the role of excess weight in undiagnosed diabetes. However, educational gradients shifted: medium education was now significantly associated with higher odds of uDM, reversing the protective pattern seen for high education in the main analysis.

When comparing uDM to diagnosed diabetes, the overall pattern persisted—undiagnosed individuals tended to be younger, female, and of better general health—but high education emerged as a significant predictor of remaining undiagnosed, a contrast to the non-significant association observed under the original cutoff.

## Discussion

### Undiagnosed diabetes in Continental Europe: prevalence and regional disparities

The study reveals that 7.2% of individuals aged 50 + across 11 European countries plus Israel (all high-income countries [28]) had undiagnosed diabetes in 2015 i.e., among all people living with diabetes, approximately 34.2% remained undiagnosed (uDM/tDM). This is comparable to 36.6% undiagnosed (aged 20–79) among people living with diabetes in European high-income countries (IDF estimates, 2013) [29]. In the SHARE population, we observe a higher uDM prevalence in Mediterranean countries, particularly Spain, Greece, and Israel, compared to northern and central Europe.

Compared to other international studies focusing on middle-aged and older adults, including China (The China Health and Retirement Longitudinal Study, CHARLS) in Asia and England (The English Longitudinal Study of Ageing, ELSA) in Europe, the uDM prevalence and the undiagnosed rate among all people living with diabetes in the SHARE sample (in 2015) is lower than in CHARLS with 59.3% representing 10.3% of the Chinese target population in 2011/12 [30], but higher than in ELSA with 22.2% representing 3.4% of the English target population in 2012/13 [31]. The lower undiagnosed rate in ELSA may suggest that the English healthcare system has more effective diabetes screening programs for the older population, enabling earlier diagnosis. Whereas the much higher undiagnosed rate in CHARLS could indicate potential barriers to healthcare access in China, such as financial constraints, lower health awareness, or insufficient routine screening among older adults. Differences in the diagnostic methodology should be also considered: ELSA and CHARLS measure HbA1c values in venous blood vs. DBS from capillary blood in SHARE. However, studies have shown that HbA1c results obtained from DBS are comparable and highly correlated with those obtained from venous samples [32].

Compared to other health surveys conducted within individual European countries, the uDM prevalence in the 2015-SHARE sample is higher: The Danish General Suburban Population Study (GESUS; 2011, age 20–100, HbA1c) reported 1.4% uDM [33] compared 4.5% uDM in SHARE; BELHES (Belgian Health Examination Survey from 2018, age ≥ 18, fasting blood glucose or HbA1c) reported 3.2% uDM [34] compared to 5.7% uDM in SHARE; DEGS (German Health Interview and Examination Survey from 2008 to 2011, age 40–79, HbA1c measurement) in Germany found 2.9% uDM [35] compared to 8.1% uDM in SHARE; the Di@bet.es Study (a National Diabetes Study from 2009 to 2011 in Spain, age ≥ 18 years, glucose tolerance test) observed 6.8% uDM [36] compared to 14.4% uDM in SHARE. Yet, SHARE measured HbA1c levels in older adults 50–100yrs. Moreover, the majority of national HES surveys were conducted before 2015. Therefore, both the older population and the increasing diabetes incidence may have driven the higher rate of uDM in the SHARE DBS countries. This comparison also highlights that due to differences in target populations, survey years, and measurement methods, cross-survey comparisons should be interpreted with care. However, a similar ranking of prevalence across countries is also observed in these national health studies, with Mediterranean countries showing the higher and northern and central Europe showing the lower prevalence of uDM.

This regional disparity in uDM prevalence closely mirrors the pattern of diabetes incidence observed in 2017 (Data from SHARE Wave 7), as indicated by longitudinal analysis of self-reported diabetes diagnoses from Waves 4 to 7 [37]. The alignment between these patterns suggests that regions with a higher burden of preDM and uDM in Wave 5 were also the regions experiencing higher diabetes incidence in Wave 7. This trend likely reflects that some of those cases identified in SHARE in 2015 (Wave 6) through blood biomarker analysis may have progressed to diagnosed diabetes (dDM) by 2017 (Wave 7).

**Fig. 4:**
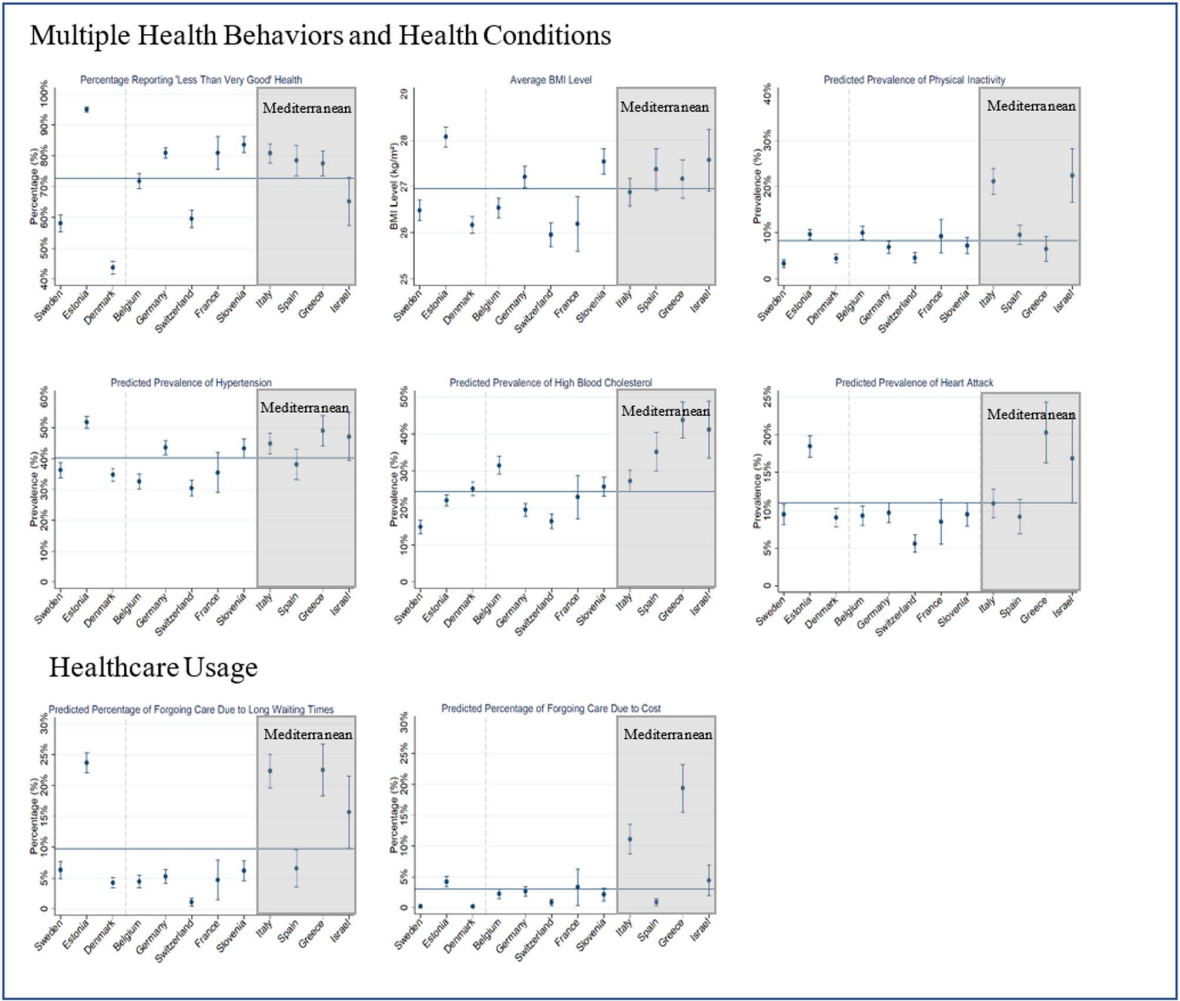
Country-level difference in health behaviors, health conditions, and healthcare usage Data Source: The Survey of Health, Ageing, and Retirement in Europe, Wave 6 in 2015 Note: Northern EU: Sweden, Estonia, Denmark; Central EU**:** Belgium, Germany, Switzerland, France, Slovenia; Mediterranean countries: Italy, Spain, Greece, Israel. The horizontal blue line represents the average value across countries. Predicted prevalence values are derived from weighted logistic regression models. Country-specific DBS weights were applied to correct for sample selection bias and generate population-level estimates

The disparities in the prevalence of uDM across countries can be associated with differences in health behaviors and/or health conditions, as well as in the effectiveness and cost of the healthcare system. Figure 4compares the country-level variations in health behaviors, health conditions, and healthcare usage using data from SHARE. Compared to northern and central European countries, Mediterranean countries (Italy, Spain, Greece, and Israel) show poorer health behaviors (e.g., higher proportion of physical inactivity and higher BMI levels) and health conditions (including a higher prevalence of diabetes-related chronic diseases like hypertension, high blood cholesterol) and worse self-rated health. Additionally, these countries face greater demands on their healthcare systems, with a significant number of individuals forgoing healthcare services due to high costs or long waiting times. Although geographically part of northern Europe, Estonia – with respect to health behaviors and health conditions, as well as healthcare system - aligns more closely with some Mediterranean countries. Estonia and Israel show lower undiagnosed rates among diabetics (uDM/tDM) despite having higher prevalence of diabetes and diabetes-related complications. We speculate that the higher rates of diabetes-related complications in these countries may also contribute to the earlier detection and diagnosis of diabetes. However, further investigation is needed to validate this assumption. Moreover, Israel’s population has a very diverse ethnic background, differing from the other SHARE countries with more homogenous population composition. Some of the ethnic groups in Israel have higher genetic predispositions to diabetes. Studies in Israel have shown that the Arabic population has a higher risk for diabetes than the Jewish population [38]. Genetic variation may also contribute to the differences between populations in northern and southern Europe [39], but SHARE has not collected own genetic data to confirm this.

Beyond individual-level factors, disparities in uDM prevalence and the share of undiagnosed among individuals with diabetes (uDM/tDM) may also reflect systemic differences in healthcare capacity, access, and financing. While SHARE does not include structural health system indicators, we supplement our discussion with OECD and World Bank data at the country level. Mediterranean countries such as Italy and Greece—which exhibit both high overall diabetes prevalence (dDM + uDM) and high uDM/tDM ratios—also report relatively low healthcare spending as a share of GDP, low general practitioner (GP) density, and high out-of-pocket (OOP) expenditures (see Supplementary eFigs. 1, 2 and 3) These countries also face a high burden of other chronic conditions (see Fig. 4), which may lead to a “crowding-out” effect, where limited resources are directed toward acute or advanced care at the expense of preventive services such as diabetes screening. The high proportion of high out-of-pocket spending further indicates greater financial barriers to timely healthcare access. In contrast, Switzerland and France display lower total diabetes prevalence but relatively high uDM/tDM ratios. Both countries report high healthcare spending and greater specialist density. This suggests that high undiagnosed rates are not solely a consequence of limited healthcare resources, but may also result from inefficient strategies to identify individuals who do not yet exhibit symptoms.

### Risk factors associated with uDM

When comparing individuals living unknowingly with diabetes (uDM cases) with individuals without diabetes, our finding underscores that socio-economic factors, particularly education level, significantly influence the development of uDM; individuals of lower educational backgrounds are more likely to have uDM, which is in line with existing literature [40, 41]. Obesity and hypertension are confirmed as strong predictors of uDM, demonstrating the need for targeted health interventions in populations exhibiting these risk factors. As undiagnosed individuals show similar health behaviors and self-rate their health like people without diabetes, the similarities in self-perception and behavior may contribute to not being diagnosed as the individuals do not recognize their vulnerability to diabetes. This underscores the critical role of healthcare systems in identifying and diagnosing uDM cases, as self-awareness alone may not be sufficient for early detection. It is important to note that more than half of the SHARE population had already reached the prediabetic state. Therefore, promoting educational campaigns on diabetes risk factors in already younger age groups and strengthening systematic screening programs should be key components of a comprehensive public health strategy.

Within the diabetic population, our finding reveals no significant educational differences between undiagnosed (uDM) and diagnosed diabetes (dDM), consistent with studies from Germany [35], Ireland [42], and China [30]. However, studies from England [31] and France [40] indicate that lower educational attainment increase the likelihood of remaining undiagnosed. Compared to the dDM group, those in the uDM category are younger, have healthier lifestyles and better general health, and rate themselves as healthier. However, these associations should not be interpreted as causal. One possible explanation is that individuals with healthier lifestyles (e.g., normal BMI, regular physical activity) may underestimate their personal risk and are therefore less likely to undergo screening; alternatively, a reverse causality mechanism is plausible: since diabetes develops gradually, individuals with uDM may be at an earlier stage of the disease (the majority of the indicative A1c-level was around 6.5–7%)—prior to the onset of symptoms or complications—and thus less likely to be diagnosed. Given the cross-sectional nature of our data, we are unable to disentangle these mechanisms or account for disease duration. Nonetheless, regardless of the underlying explanation, relying solely on traditional risk-factor-based screening may leave many older adults unaware of their condition. Without timely detection and lifestyle changes, many individuals with prediabetes will eventually progress to full diabetes and those with uDM may progressively develop more serious diabetes symptoms or even complications such as retinopathy, CVD, or kidney disease. Therefore, enhancing diabetes-risk communication, expanding population screenings, and providing testing incentives could raise awareness and offer early intervention opportunities. Community-based screening, particularly for hard-to-reach populations with unfavorable risk profiles, could complement clinical-based screening systems effectively [43]. The significant association between hypertension and uDM supports the strategy of combined testing for both as an effective approach to early disease detection [44].

The BMI-stratified analysis highlights that the protective effect of education is more pronounced among individuals with lower metabolic risk, particularly those with normal BMI. This pattern disappears among overweight and obese individuals, where metabolic and health-related conditions become the dominant correlates of undiagnosed diabetes. This highlights the need for more targeted screening efforts that go beyond educational attainment, especially in populations with high metabolic burden.

Under a stricter diagnostic threshold (HbA1c ≥ 7.0%), the educational gradient reverses: individuals with medium education have higher odds of having undiagnosed diabetes (vs. normoglycemic), and both medium- and high-educated groups show increased odds of remaining undiagnosed (vs. diagnosed). This likely reflects a reclassification effect, whereby lower-educated individuals near the original 6.5% cutoff are reclassified as prediabetic, while higher-educated individuals with substantially elevated HbA1c levels remain undiagnosed. One possible explanation is that highly educated individuals may have fewer metabolic risk factors or a stronger belief in being healthy leading to delayed detection despite worse glycemic control. These findings reinforce the need for screening strategies that go beyond educational attainment. Whether these associations reflect underlying causal mechanisms warrants further investigation.

### Strength and limitation

To the best of our knowledge, this study is the first harmonized and therefore comparable collection of DBS samples for blood biomarker assessment across multiple European countries. This enables cross-national comparison of blood values [18]. Easy measurement of the diabetes marker HbA1c from DBS, a minimally invasive method, allows assessment of pre- and undiagnosed diabetes cases in a population survey, which is not only highly important for individuals but also for entire national health systems. The comparison of the SHARE results with existing surveys supports the use of HbA1c measurement in population surveys for understanding the diabetes status of a country.

Limitations of our study should be noted. Firstly, the DBS derived data is cross-sectional and was only collected once in Wave 6, 2015. As the diabetes status was assessed using a single HbA1c test, multiple testing and different tests are imperative for a clinical diabetes diagnosis [45]. Therefore, the prevalence of preDM and uDM may be less precise. Secondly, the dDM is based on respondents’ self-reports, and reporting errors by the respondents as well as the interviewers may lead to some measurement error, though Mose et al. (2023) using Danish SHARE data showed that self-reported diabetes and related medications in SHARE was very reliable [46]. Thirdly, our focus is on Type 2 diabetes. With the data available in the SHARE sample, we cannot distinguish between Type 2 and other types of diabetes, such as Type 1, which comprises about 5–10% of all diabetes cases and is usually diagnosed in childhood or adolescence [47]. However, in a SHARE subgroup of respondents who recalled their age at diagnosis, only 1.8% was diagnosed before age 25 and 3.5% before age 30, suggesting a low proportion of Type 1 diabetes in the SHARE sample. Finally, while we controlled for a broad set of covariates in our models, some factors - such as family history of diabetes, risk perception (e.g., overconfidence), and health literacy - were not available in the dataset. These unobserved confounders may affect both diabetes risk and the likelihood of diagnosis. Given the cross-sectional nature of our study, we are limited to identifying associations and cannot establish a causal relationship.

The data used in this analysis were collected in 2015, but still remain relevant in the recent context, as diabetes continues to pose a major public health challenge. While the global rate of undiagnosed diabetes remained stable at 45–50% from 2013 to 2021 - with a modest decline in high-income EU countries from 36.6 to 31.1% - the overall rise in diabetes prevalence over the past decade has led to an increase in the absolute number of undiagnosed cases [29, 48]. Moreover, using self-reported diagnosis data from SHARE Wave 9 confirms that dDM in older adults aged 50 + rose from 14% in 2015 to 15.9% in 2021. Additionally, the prevalence of two key risk factors - obesity and physical inactivity - also slightly increased, to (20–21%) and (13–14%), respectively.

### Conclusion

Our findings highlight the substantial burden of undiagnosed diabetes among older adults across European countries; identifying individuals with uDM is essential for accurately estimating total diabetes prevalence and for informing early diagnostic efforts, particularly considering that more than half of our study population is in preDM and uDM stages. By applying a harmonized methodology, we observed notable regional disparities, with Mediterranean countries such as Italy and Greece exhibiting higher prevalence of both dDM and uDM. Our findings provide important insights for public health policies and diabetes screening strategies.

### Future research potential

Future research could examine how healthcare system structure and capacity influence diabetes diagnosis, and investigate specific barriers that lead to under-screening of diabetes to increase the efficiency of the screening system and the detection process. In addition, since our findings are based on cross-sectional data, future studies should consider using longitudinal data to investigate the causal relationships between risk factors and diabetes diagnosis.

## Supplementary Information

Supplementary Material 1.

## Data Availability

The dataset utilized in this study is based on publicly available data from the Survey of Health, Ageing, and Retirement in Europe (SHARE, Wave 6, 2015), a cross-national population survey covering multiple European countries and Israel. SHARE data are available for scientific research upon request. Researchers can apply for access by completing and signing the SHARE User Statement, which outlines the conditions of use: (see in https://share-eric.eu/data/data-access/conditions-of-use). Once approved, the data can be downloaded from the SHARE Research Data Center: https://share-eric.eu/data.

## Abbreviations

uDM: Undiagnosed Diabetes Mellitus
dDM: Diagnosed Diabetes Mellitus
tDM: Total Diabetes Mellitus
preDM: Prediabetes Mellitus
DBS: Dried Blood Spots
SHARE: Survey of Health, Ageing, and Retirement in Europe
CHARLS: The China Health and Retirement Longitudinal Study
ELSA: The English Longitudinal Study of Ageing
SES: Socio–Economic Status
IDF: International Diabetes Foundation
VB: Venous Blood
HbA1c: Glycated Hemoglobin
CVD: Cardiovascular Disease
BMI: Body Mass Index
ISCED: International Standard Classification of Education
STATA: Software for statistical analysis
OR: Odds Ratio
OOP: Out–Of–Pocket

## References

[1] International Diabetes Federation. IDF Diabetes Atlas. 10th ed. 2021.

[2] Neupane S, Florkowski WJ, Dhakal U, Dhakal C. Regional disparities in type 2 diabetes prevalence and associated risk factors in the united States. Diabetes Obes Metab. 2024;26(10):4776–82. 10.1111/dom.15797. 39021356 10.1111/dom.15797

[3] International Diabetes Federation. IDF Diabetes Atlas. 7th ed. 2015.

[4] Gudjonsdottir H, Tynelius P, Stattin NS, Méndez DY, Lager A, Brynedal B. Undiagnosed type 2 diabetes is common–intensified screening of established risk groups is imperative in sweden: the SDPP cohort. BMC Med. 2024;22(1):168. 10.1186/s12916-024-03393-0. 38637767 10.1186/s12916-024-03393-0 PMC11027361

[5] Gopalan A, Mishra P, Alexeeff SE, et al. Prevalence and predictors of delayed clinical diagnosis of type 2 diabetes: a longitudinal cohort study. Diabet Med. 2018;35(12):1655–62. 10.1111/dme.13808. 30175870 10.1111/dme.13808 PMC6481650

[6] Lyu F, Wu D, Wei C, Wu A. Vascular cognitive impairment and dementia in type 2 diabetes mellitus: an overview. Life Sci. 2020;254:117771. 10.1016/j.lfs.2020.117771. 32437791 10.1016/j.lfs.2020.117771

[7] Wu K, Liu H, Zheng J, Zou L, Gu S, Zhou R, et al. Diabetes treatment is associated with better cognitive function: the age disparity. Front Aging Neurosci. 2022;13:753129. 10.3389/fnagi.2021.753129. 35069170 10.3389/fnagi.2021.753129 PMC8770273

[8] Verma A, Vyas S, Agarwal A, Abbas S, Agarwal DP, Kumar R. Diabetic kidney disease and hypertension: a true love story. J Clin Diagn Research: JCDR. 2016;10(3):OC11. 10.7860/JCDR/2016/18806.7511. 10.7860/JCDR/2016/18806.7511 PMC4843298 27134912

[9] Ruigómez A, García Rodríguez LA. Presence of diabetes related complication at the time of NIDDM diagnosis: an important prognostic factor. Eur J Epidemiol. 1998;14:439–45. 10.1023/A:1007484920656. 9744675 10.1023/a:1007484920656

[10] Dall TM, Yang W, Gillespie K, Mocarski M, Byrne E, Cintina I, Beronja K, Semilla AP, Iacobucci W, Hogan PF. The economic burden of elevated blood glucose levels in 2017: diagnosed and undiagnosed diabetes, gestational diabetes mellitus, and prediabetes. Diabetes Care. 2019;42(9):1661–8. 10.2337/dc18-1226. 30940641 10.2337/dc18-1226 PMC6702607

[11] Sortsø C, Komkova A, Sandbæk A, et al. Effect of screening for type 2 diabetes on healthcare costs: a register-based study among 139,075 individuals diagnosed with diabetes in Denmark between 2001 and 2009. Diabetologia. 2018;61(6):1306–14. 10.1007/s00125-018-4594-2. 29549417 10.1007/s00125-018-4594-2 PMC7617192

[12] Butler JS, Burkhauser RV, Mitchell JM, Pincus TP. Measurement error in self-reported health variables. Rev Econ Stat. 1987;69:644–50. 10.2307/1935959.

[13] Heiss F, McFadden D, Winter J, Wuppermann A, Zhu Y. Measuring disease prevalence in surveys. Insights Econ Aging Univ Chic Press. 2017;227–52. 10.7208/chicago/9780226426709.001.0001.

[14] Wolinsky FD, Jones MP, Ullrich F, Lou Y, Wehby GL. The concordance of survey reports and medicare claims in a nationally representative longitudinal cohort of older adults. Med Care. 2014;52:462–8. 10.1097/MLR.0000000000000120. 24714584 10.1097/MLR.0000000000000120

[15] Kim JK, Faul J, Weir DR, Crimmins EM. Dried blood spot based biomarkers in the health and retirement study: 2006 to 2016. Am J Hum Biol. 2024;36:e23997. 10.1002/ajhb.23997. 37803815 10.1002/ajhb.23997 PMC10873048

[16] Steptoe A, Breeze E, Banks J, Nazroo J. Cohort profile: the english longitudinal study of ageing. Int J Epidemiol. 2013;42:1640–8. 10.1093/ije/dys168. 23143611 10.1093/ije/dys168 PMC3900867

[17] Kearney PM, Cronin H, O’Regan C, Kamiya Y, Savva GM, Whelan B, et al. Cohort profile: the Irish longitudinal study on ageing. Int J Epidemiol. 2011;40:877–84. 10.1093/ije/dyr116. 21810894 10.1093/ije/dyr116

[18] Börsch-Supan M, Horton HM, Sun A, Weiss L, Groh R, Schmidutz D et al. Biomarkers in SHARE: documentation of implementation, collection, and analysis of dried blood spot (DBS) samples 2015–2023. NBER Working Paper. 2024;(w32764). 10.3386/w32764.

[19] Zhao Y, Hu Y, Smith JP, Strauss J, Yang G. Cohort profile: the China health and retirement longitudinal study (CHARLS). Int J Epidemiol. 2014;43:61–8. 10.1093/ije/dys203. 23243115 10.1093/ije/dys203 PMC3937970

[20] Tolonen H, Koponen P, Al-kerwi A, Capkova N, Giampaoli S, Mindell J, et al. European health examination surveys–a tool for collecting objective information about the health of the population. Arch Public Health. 2018;76:38. 10.1186/s13690-018-0282-4. 29988297 10.1186/s13690-018-0282-4 PMC6022327

[21] Bowen CL, Evans CA. Challenges and experiences with dried blood spot technology for method development and validation. Dried Blood Spots: Appl Techniques. 2014;27:179–87. 10.1002/9781118890837.ch15.

[22] Thomas D, Seeman T, Potter A, Hu P, Crimmins E, Herningtyas EH, Sumantri C, Frankenberg E. HPLC-based measurement of glycated hemoglobin using dried blood spots collected under adverse field conditions. Biodemography Social Biology. 2018;64(1):43–62. 10.1080/19485565.2018.1451300. 29741414 10.1080/19485565.2018.1451300 PMC6173327

[23] Hu P, Crimmins EM, Kim JK, Potter A, Cofferen J, Merkin S, McCreath H, Seeman T. Harmonization of four biomarkers across nine nationally representative studies of older persons. Am J Hum Biology. 2024;36(5):e24030. 10.1002/ajhb.24030. 10.1002/ajhb.24030 PMC11062831 38069621

[24] Börsch-Supan A, Weiss LM, Börsch‐Supan M, Potter AJ, Cofferen J, Kerschner E. Dried blood spot collection, sample quality, and fieldwork conditions: structural validations for conversion into standard values. Am J Hum Biology. 2021;33(4):e23517. 10.1002/ajhb.23517. 10.1002/ajhb.23517 PMC10980534 33063418

[25] Börsch-Supan A, Brandt M, Hunkler C, Kneip T, Korbmacher J, Malter F, et al. Data resource profile: the survey of health, ageing and retirement in Europe (SHARE). Int J Epidemiol. 2013;42:992–1001. 10.1093/ije/dyt088. 23778574 10.1093/ije/dyt088 PMC3780997

[26] Nathan DM, Turgeon H, Regan S. Relationship between glycated haemoglobin levels and mean glucose levels over time. Diabetologia. 2007;50:2239–44. 10.1007/s00125-007-0803-0. 17851648 10.1007/s00125-007-0803-0 PMC8752566

[27] American Diabetes Association. Diagnosis and classification of diabetes mellitus. Diabetes Care. 2013;36(Supplement1):S67–74. 10.2337/dc13-S067. 23264425 10.2337/dc13-S067 PMC3537273

[28] The World Bank. The World by Income and Region in 2013. https://datatopics.worldbank.org/world-development-indicators/the-world-by-income-and-region.html. Accessed 15 Apr 2024.

[29] Beagley J, Guariguata L, Weil C, Motala AA. Global estimates of undiagnosed diabetes in adults. Diabetes Res Clin Pract. 2014;103(2):150–60. 10.1016/j.diabres.2013.11.001. 24300018 10.1016/j.diabres.2013.11.001

[30] Zhao Y, Crimmins EM, Hu P, Shen Y, Smith JP, Strauss J, Wang Y, Zhang Y. Prevalence, diagnosis, and management of diabetes mellitus among older chinese: results from the China health and retirement longitudinal study. Int J Public Health. 2016;61(3):347–56. 10.1007/s00038-015-0780-x. 26755457 10.1007/s00038-015-0780-x PMC4880519

[31] Huang YT, Steptoe A, Zaninotto P. Prevalence of undiagnosed diabetes in 2004 and 2012: evidence from the english longitudinal study of aging. Journals Gerontology: Ser A. 2021;76(5):922–8. 10.1093/gerona/glaa179. 10.1093/gerona/glaa179 PMC8522434 32674123

[32] Nathan DM, Krause-Steinrauf H, Braffett BH, Arends VL, Younes N, McGee P, et al. Comparison of central laboratory HbA1c measurements obtained from a capillary collection versus a standard venous whole blood collection in the GRADE and EDIC studies. PLoS ONE. 2021;16:e0257154. 10.1371/journal.pone.0257154. 34780485 10.1371/journal.pone.0257154 PMC8592405

[33] Jørgensen ME, Ellervik C, Ekholm O, Johansen NB, Carstensen B. Estimates of prediabetes and undiagnosed type 2 diabetes in denmark: the end of an epidemic or a diagnostic artefact? Scand J Public Health. 2020;48:106–12. 10.1177/1403494818799606. 30222048 10.1177/1403494818799606

[34] Sciensano. Non-Communicable Diseases: Diabetes, Health Status Report, Brussels, Belgium. *Sciensano.* 2023. https://www.healthybelgium.be/en/health-status/non-communicable-diseases/diabetes. Accessed 2 July 2024.

[35] Du Y, Baumert J, Paprott R, Teti A, Heidemann C, Scheidt-Nave C. Factors associated with undiagnosed type 2 diabetes in germany: results from German health interview and examination survey for adults 2008–2011. BMJ Open Diabetes Res Care. 2020;8(1):e001707. 10.1136/bmjdrc-2020-001707. 33067247 10.1136/bmjdrc-2020-001707 PMC7569997

[36] Soriguer F, Goday A, Bosch-Comas A, Bordiu E, Calle-Pascual A, Carmena R, et al. Prevalence of diabetes mellitus and impaired glucose regulation in spain: the di@bet.es study. Diabetologia. 2012;55:88–93. 10.1007/s00125-011-2336-9. 21987347 10.1007/s00125-011-2336-9 PMC3228950

[37] Elek P, Bíró A. Regional differences in diabetes across Europe–regression and causal forest analyses. Econ Hum Biol. 2021;40:100948. 10.1016/j.ehb.2020.100948. 33276258 10.1016/j.ehb.2020.100948

[38] Jaffe A, Giveon S, Wulffhart L, Oberman B, Baidousi M, Ziv A, Kalter-Leibovici O. Adult Arabs have higher risk for diabetes mellitus than Jews in Israel. PLoS ONE. 2017;12(5):e0176661. 10.1371/journal.pone.0176661. 28481942 10.1371/journal.pone.0176661 PMC5421762

[39] Tamayo T, Rosenbauer J, Wild SH, Spijkerman AMW, Baan C, Forouhi NG, et al. Diabetes in europe: an update. Diabetes Res Clin Pract. 2014;103:206–17. 10.1016/j.diabres.2013.11.007. 24300019 10.1016/j.diabres.2013.11.007

[40] Lailler G, Piffaretti C, Fuentes S, et al. Prevalence of prediabetes and undiagnosed type 2 diabetes in france: results from the National survey ESTEBAN, 2014–2016. Diabetes Res Clin Pract. 2020;165:108252. 10.1016/j.diabres.2020.108252. 32526264 10.1016/j.diabres.2020.108252

[41] Dos Santos ESM, Máximo RO, de Andrade FB, de Oliveira C, Lima-Costa MF, Alexandre TDS. Differences in the prevalence of prediabetes, undiagnosed diabetes and diagnosed diabetes and associated factors in cohorts of Brazilian and english older adults. Public Health Nutr. 2021;24(13):4187–94. 10.1017/S1368980020003201. 32972476 10.1017/S1368980020003201 PMC9014254

[42] Leahy S, O’Halloran AM, O’Leary N, Healy M, McCormack M, Kenny RA, et al. Prevalence and correlates of diagnosed and undiagnosed type 2 diabetes mellitus and pre-diabetes in older adults: findings from the Irish longitudinal study on ageing (TILDA). Diabetes Res Clin Pract. 2015;110:241–9. 10.1016/j.diabres.2015.10.015. 26520567 10.1016/j.diabres.2015.10.015

[43] Timm L, Harcke K, Karlsson I, Sidney Annerstedt K, Alvesson HM, Stattin NS, et al. Early detection of type 2 diabetes in socioeconomically disadvantaged areas in Stockholm–comparing reach of community and facility-based screening. Glob Health Action. 2020;13:1795439. 10.1080/16549716.2020.1795439. 32746747 10.1080/16549716.2020.1795439 PMC7480601

[44] Di Girolamo C, Turatto F, Destefanis C, Formenti B, Armocida B, Lionello L, et al. Joint Action JACARDI: a context analysis on CVDs and diabetes policies and practices. Eur J Public Health. 2024;34(Suppl 3):ckae144–1281. 10.1093/eurpub/ckae144.1281.

[45] Lipska KJ, Inzucchi SE, Van Ness PH, Gill TM, Kanaya A, Strotmeyer ES, et al. Elevated HbA1c and fasting plasma glucose in predicting diabetes incidence among older adults: are two better than one? Diabetes Care. 2013;36:3923–9. 10.2337/dc12-2631. 24135387 10.2337/dc12-2631 PMC3836095

[46] Mose J, Jensen KH, Scheel-Hincke LL, Andersen-Ranberg K. Are self-reported medical conditions and medicine use from middle-aged and older adults credible? A validation study comparing Danish SHARE-data with National health register data. Ann Epidemiol. 2023;87:100–6. 10.1016/j.annepidem.2023.09.009. 37903678 10.1016/j.annepidem.2023.09.009

[47] DiMeglio LA, Evans-Molina C, Oram RA. Type 1 diabetes. Lancet. 2018;391(10138):2449–62. 10.1016/S0140-6736(18)31320-5. 29916386 10.1016/S0140-6736(18)31320-5 PMC6661119

[48] Ogurtsova K, Guariguata L, Barengo NC, Lopez-Doriga Ruiz P, Sacre JW, Karuranga S, et al. IDF diabetes atlas: global estimates of undiagnosed diabetes in adults for 2021. Diabetes Res Clin Pract. 2022;183:109118. 10.1016/j.diabres.2021.109118. 34883189 10.1016/j.diabres.2021.109118

[49] World Medical Association. World medical association declaration of helsinki: ethical principles for medical research involving human subjects. JAMA. 2013;310(20):2191–4. 24141714 10.1001/jama.2013.281053

