# Supplementary material for: Undiagnosed diabetes mellitus among older adults: a harmonized cross-country analysis of prevalence, regional disparities and risk factors in 11 European countries and Israel
Source: BMC Public Health. 2025 Aug 8;25:2705. doi: 10.1186/s12889-025-23766-1 (PMC12333259; doi:10.1186/s12889-025-23766-1)
Supplement: Supplementary file 1 — Supplementary Material 1. [file 12889_2025_23766_MOESM1_ESM.docx]

**Supplement**

eTable 1: Comparison of characteristics between DBS collection* and Non-DBS collection regions in France

| **Variable Name** | **Region with DBS Collection (Mean)** | **Region with Non-DBS Collection (Mean)** | **Significance Level**  **(p-value)** |
| --- | --- | --- | --- |
| Age | 67.648 | 68.428 | 0.221 |
| Gender | 0.431 | 0.428 | 0.916 |
| Education Group | 1.812 | 1.871 | 0.196 |
| Diagnosed diabetes | 0.122 | 0.122 | 0.998 |
| Hypertension | 0.322 | 0.379 | 0.038* |
| High Cholesterol | 0.224 | 0.238 | 0.578 |
| Stroke | 0.040 | 0.026 | 0.218 |
| Chronic Kidney Disease | 0.018 | 0.019 | 0.846 |
| Forgo care - cost | 0.029 | 0.026 | 0.744 |
| Forgo care - long wait | 0.069 | 0.045 | 0.103 |
| * DBS collection was implemented in four districts of France: Aquitaine, Bourgogne, Bretagne, and Île-de-France. | | | |

eTable 2: Prevalence of undiagnosed and diagnosed diabetes, and the undiagnosed rate among individuals with diabetes (uDM/tDM), by age and gender

|  | **50-59** | **60-69** | **70-79** | **80+** |
| --- | --- | --- | --- | --- |
| **Female** |  |  |  |  |
| Undiagnosed diabetes % | 9.3 | 9.7 | 8.6 | 9.8 |
| 95% CI | [6.2 - 12.3] | [7.7 - 11.6] | [6.5 - 10.7] | [6.7 - 12.9] |
| Diagnosed diabetes % | 6.7 | 12.1 | 17.6 | 18.5 |
| 95% CI | [4.6 - 8.9] | [10.1 - 14.1] | [14.8 - 20.4] | [14.3 - 22.7] |
| Undiagnosed among individuals with diabetes % | 58.7 | 44.3 | 32.3 | 35.0 |
| 95% CI | [47.4 - 69.9] | [37.6 - 51.1] | [25.7 - 38.8] | [25.3 - 44.8] |
| **Men** |  |  |  |  |
| Undiagnosed diabetes % | 10.6 | 7.9 | 8.2 | 8.8 |
| 95% CI | [6.5 - 14.8] | [5.7 - 10] | [6 - 10.3] | [5.6 - 12.1] |
| Diagnosed diabetes % | 12.3 | 18.5 | 22.8 | 23.6 |
| 95% CI | [9.1 - 15.6] | [15.4 - 21.6] | [19.2 - 26.4] | [18.6 - 28.5] |
| Undiagnosed among individuals with diabetes % | 47.1 | 29.2 | 25.7 | 27.5 |
| 95% CI | [35.4 - 58.8] | [22.1 - 36.2] | [19.4 - 32.0] | [18.6 - 36.4] |
| * Results are weighted to represent the prevalence in the population. |  |  |  |  |

eTable 3: The estimated prevalence rates of prediabetes, undiagnosed, diagnosed , total diabetes, and the rate of diabetes diagnosis across 11 European countries and Israel (in %)

| **Country** | **preDM** | | **uDM** | | **dDM** | | **tDM** | | **uDM / tDM** | |
| --- | --- | --- | --- | --- | --- | --- | --- | --- | --- | --- |
|  | Unadjusted | Unadjusted | Adjusted | Unadjusted | Unadjusted | Adjusted | Unadjusted | Adjusted | Unadjusted | Adjusted |
| Sweden | 56.4 | 55.8 | 6.6 | 7.0 | 11.5 | 11.9 | 18.2 | 18.9 | 36.5 | 36.4 |
|  | [53.7 - 59.1] | [53.0 - 58.6] | [5.2 - 8.0] | [5.5 - 8.4] | [9.9 - 13.2] | [10.2 - 13.6] | [16.0 - 20.2] | [16.7 - 21.1] | [30.1 - 42.5] | [29.9 - 42.5] |
| Estonia | 58.2 | 56.8 | 5.0 | 5.1 | 15.8 | 15.8 | 20.8 | 20.9 | 23.9 | 23.8 |
|  | [56.4 - 60.2] | [54.9 - 59.1] | [4.1 - 5.8] | [4.2 - 6.0] | [14.5 -17.2] | [14.3 - 17.3] | [19.2 - 22.3] | [19.3 - 22.6] | [20.2 - 27.3] | [19.7 - 27.5] |
| Denmark | 52.0 | 50.9 | 4.5 | 5.0 | 7.9 | 9.0 | 12.4 | 14 | 36.4 | 36.2 |
|  | [50.1 - 54.2] | [48.6 - 53.3] | [3.7 - 5.4] | [4.0 - 6.0] | [6.8 - 9.0] | [7.7- 10.3] | [11.1 - 13.8] | [12.4 - 15.6] | [30.8 - 41.9] | [30.1 - 42.3] |
| Belgium | 54.5 | 54.3 | 5.7 | 6.0 | 12.3 | 13.2 | 18.1 | 19.2 | 31.8 | 31.3 |
|  | [51.9 - 57.0] | [51.6 - 56.8] | [4.7 - 6.8] | [4.9 - 7.1] | [10.8 - 13.8] | [11.6 - 14.8] | [16.3 - 19.9] | [17.3 - 21.0] | [26.9 - 36.6] | [26.4 - 36.2] |
| Germany | 53.6 | 52.6 | 8.1 | 8.5 | 16.1 | 17.4 | 24.2 | 26.0 | 33.6 | 32.5 |
|  | [51.4 - 55.9] | [50.2 - 55.3] | [6.8 - 9.4] | [7.0 - 10.1] | [14.4 - 17.8] | [15.3 - 19.5] | [22.2 - 26.2] | [23.5 - 28.4] | [29.1 - 38.1] | [27.5 - 37.4] |
| Switzerland | 59.6 | 59.1 | 5.4 | 5.4 | 8.2 | 8.3 | 13.5 | 13.6 | 39.6 | 41.6 |
|  | [56.9 - 62.4] | [56.3 - 62.2] | [4.2 - 6.5] | [4.1 - 6.7] | [6.8 - 9.6] | [6.8 - 9.8] | [11.7 - 15.3] | [11.8 - 15.5] | [32.7 - 46.4] | [34.2 - 49.0] |
| France | 48.9 | 49.0 | 8.1 | 8.3 | 9.5 | 10.3 | 17.6 | 18.6 | 46.1 | 47.9 |
|  | [42.3 - 56.1] | [42.6 - 56.0] | [4.2 - 12.1] | [4.3 - 12.2] | [6.3 - 12.7] | [71 - 3.6] | [12.8 - 22.5] | [13.7 - 23.5] | [30.9 - 61.3] | [34.7 - 61.1] |
| Slovenia | 56.1 | 56.1 | 9.7 | 9.6 | 15.5 | 16.3 | 25.2 | 25.8 | 38.6 | 36.8 |
|  | [53.1 - 59.3] | [53.1 - 59.5] | [8.0 - 11.6] | [7.8 - 11.4] | [13.3 - 17.6] | [14.0 - 18.5] | [22.6 - 27.9] | [23.1 - 28.6] | [32.9 - 44.6] | [31.1 - 42.7] |
| Italy | 50.7 | 51.6 | 9.9 | 9.3 | 15.7 | 13.5 | 25.6 | 22.8 | 38.7 | 40.7 |
|  | [47.1 - 54.0] | [47.6 - 55.1] | [7.7 - 12.1] | [7.0 - 11.5] | [13.3 - 18.0] | [11.3 - 15.7] | [22.6 - 28.6] | [19.9 - 25.7] | [31.9 - 45.6] | [34.0 - 47.5] |
| Spain | 46.8 | 48.1 | 14.4 | 13.4 | 20.9 | 19.6 | 35.2 | 33.0 | 40.8 | 38.4 |
|  | [40.9 - 51.3] | [42.0 - 52.8] | [10.6 - 18.1] | [9.7 - 17.1] | [16.7 - 25.0] | [15.4 - 23.8] | [30.3 - 40.2] | [28.0 - 38.0] | [32.1 - 49.5] | [29.7 - 47.1] |
| Greece | 51.1 | 51.0 | 15.1 | 14.8 | 22.0 | 18.8 | 37.1 | 33.6 | 40.6 | 44.2 |
|  | [46.3 - 56.0] | [46.0 - 56.1] | [11.7 - 18.4] | [11.3 - 18.3] | [17.9 - 26.2] | [15.0 - 22.6] | [32.4 - 41.8] | [29.1 - 38.1] | [32.9 - 48.4] | [35.7 - 52.7] |
| Israel | 24.6 | 24.5 | 9.9 | 10.1 | 33.8 | 32.9 | 43.6 | 43.0 | 22.6 | 22.4 |
|  | [19.0 - 29.9] | [19.1 - 29.5] | [3.2 - 16.6] | [3.3 - 16.9] | [25.4 - 42.1] | [24.6 - 41.2] | [35.1 - 52.1] | [34.6 - 51.5] | [8.5 - 36.7] | [8.6 - 36.3] |
| Source: The Survey of Health, Ageing, and Retirement in Europe, Wave 6 in 2015, 11 European countries and Israel  Note: preDM: prediabetes mellitus, uDM: undiagnosed diabetes mellitus, dDM: diagnosed diabetes mellitus, tDM: total diabetes mellitus, encompassing both undiagnosed and diagnosed diabetes mellitus. The unadjusted prevalence rates were estimated based on the existing demographic characteristics of the country. Adjusted prevalence rates were calculated by standardizing for age, gender, and education levels to the average levels observed across multiple countries, thereby holding these factors constant across different populations. | | | | | | | | | | |

eTable 4: Sample characteristics across 11 Europeans countries and Israel

| **Baseline Characteristic** | Total | Sweden | Estonia | Denmark | Belgium | Germany | Switzerland | France | Slovenia | Italy | Spain | Greece | Israel |
| --- | --- | --- | --- | --- | --- | --- | --- | --- | --- | --- | --- | --- | --- |
| **N** | 19133 | 2065 | 3131 | 2449 | 2540 | 2419 | 1565 | 301 | 1558 | 1297 | 962 | 472 | 374 |
| **Gender** |  |  |  |  |  |  |  |  |  |  |  |  |  |
| Male | 42.7 | 44.5 | 33.9 | 44.9 | 43.9 | 46.9 | 47.0 | 42.6 | 41.6 | 44.5 | 40.0 | 44.1 | 44.4 |
| **Age Group** |  |  |  |  |  |  |  |  |  |  |  |  |  |
| Age (mean, years) | 67.8 | 69.5 | 70.3 | 65.5 | 66.1 | 65.8 | 68.3 | 68.5 | 67.3 | 67.9 | 69.0 | 69.6 | 70.8 |
| 50-59 | 22.2 | 12.8 | 15.1 | 31.3 | 29.2 | 29.4 | 20.6 | 22.0 | 23.6 | 21.2 | 19.2 | 9.7 | 7.8 |
| 60-69 | 36.8 | 39.5 | 32.6 | 37.4 | 38.5 | 35.5 | 37.7 | 36.1 | 36.4 | 36.3 | 35.4 | 45.2 | 43.4 |
| 70-79 | 28.1 | 34.0 | 33.9 | 22.4 | 21.2 | 27.3 | 27.3 | 24.9 | 27.3 | 31.5 | 28.4 | 28.0 | 30.2 |
| >=80 | 13.0 | 13.7 | 18.4 | 8.9 | 11.1 | 7.8 | 14.4 | 17.0 | 12.7 | 11.0 | 17.0 | 17.1 | 18.5 |
| **Immigration** |  |  |  |  |  |  |  |  |  |  |  |  |  |
| Yes | 11.3 | 7.8 | 21.1 | 3.2 | 7.8 | 12.1 | 12.1 | 8.6 | 16.8 | 1.8 | 2.5 | 2.5 | 60.3 |
| **Cohabitation** |  |  |  |  |  |  |  |  |  |  |  |  |  |
| Yes | 67.6 | 68.0 | 53.7 | 70.1 | 65.2 | 73.4 | 70.3 | 63.5 | 66.3 | 78.1 | 74.7 | 77.4 | 72.6 |
| **ISCED Group** |  |  |  |  |  |  |  |  |  |  |  |  |  |
| Years of Education  (mean, years) | 11.4 | 11.8 | 11.7 | 13.6 | 12.7 | 12.7 | 8.6 | 12.1 | 10.4 | 8.6 | 7.9 | 9.0 | 12.6 |
| Low Edu | 33.1 | 34.3 | 28.3 | 17.0 | 38.4 | 11.0 | 20.2 | 39.3 | 33.3 | 71.7 | 80.8 | 57.1 | 36.6 |
| Medium Edu | 40.2 | 33.9 | 49.1 | 37.9 | 26.4 | 57.2 | 63.2 | 34.4 | 49.9 | 21.7 | 10.1 | 26.4 | 26.8 |
| High Edu | 26.8 | 31.7 | 22.7 | 45.0 | 35.2 | 31.9 | 16.7 | 26.2 | 16.8 | 6.6 | 9.2 | 16.5 | 36.6 |
| **Income Quartiles** |  |  |  |  |  |  |  |  |  |  |  |  |  |
| Household Income  (mean, euro) | 35992 | 41577 | 8424 | 46844 | 52471 | 34203 | 92011 | 35140 | 16011 | 22702 | 17967 | 13177 | 34339 |
| 1st quantiles | 25.2 | 3.4 | 83.5 | 3.6 | 4.6 | 8.8 | 2.1 | 7.9 | 44.9 | 19.8 | 35.8 | 61.4 | 19.3 |
| 2nd quantiles | 24.9 | 19.8 | 14.4 | 19.0 | 27.3 | 28.6 | 4.5 | 30.5 | 40.7 | 47.1 | 44.3 | 26.4 | 26.1 |
| 3rd quantiles | 25.0 | 39.8 | 1.9 | 33.1 | 33.4 | 39.6 | 20.1 | 38.7 | 11.6 | 26.8 | 16.5 | 10.1 | 32.2 |
| 4th quantiles | 24.9 | 36.9 | 0.2 | 44.3 | 34.8 | 23.1 | 73.3 | 23.0 | 2.8 | 6.4 | 3.4 | 2.1 | 22.4 |
| **Health Behaviors** |  |  |  |  |  |  |  |  |  |  |  |  |  |
| Physical Inactivity | 8.5 | 4.0 | 9.9 | 4.2 | 9.8 | 4.9 | 4.4 | 10.8 | 7.4 | 20.9 | 14.4 | 5.6 | 26.1 |
| Ever Smoked | 47.1 | 55.1 | 40.3 | 58.6 | 49.8 | 48.6 | 46.9 | 40.7 | 38.3 | 43.3 | 40.0 | 47.0 | 32.2 |
| Non-drinkers | 78.4 | 79.0 | 82.8 | 60.9 | 71.8 | 73.2 | 88.5 | 85.7 | 78.3 | 93.1 | 93.7 | 78.1 | 92.5 |
| Moderate drinkers | 15.8 | 15.1 | 13.7 | 32.9 | 17.7 | 19.1 | 9.4 | 12.3 | 14.2 | 2.5 | 3.3 | 18.2 | 4.0 |
| Frequent drinkers | 5.8 | 5.9 | 3.5 | 6.2 | 10.5 | 7.8 | 2.1 | 2.0 | 7.6 | 4.4 | 3.0 | 3.7 | 3.5 |
| **Self-reported Health** |  |  |  |  |  |  |  |  |  |  |  |  |  |
| Very good | 26.1 | 23.0 | 57.4 | 4.4 | 38.9 | 26.7 | 19.9 | 27.3 | 17.9 | 12.9 | 18.3 | 38.6 | 19.9 |
| Good | 35.3 | 35.8 | 22.2 | 20.1 | 35.5 | 26.9 | 42.1 | 47.1 | 37.8 | 44.6 | 40.4 | 42.2 | 45.8 |
| Fair/Poor | 38.6 | 41.2 | 20.4 | 75.5 | 25.6 | 46.4 | 38.0 | 25.7 | 44.4 | 42.5 | 41.3 | 19.2 | 34.2 |
| **To be continued on next page** | | | | | | | | | | | | | |
| **Baseline Characteristic** | Total | Sweden | Estonia | Denmark | Belgium | Germany | Switzerland | France | Slovenia | Italy | Spain | Greece | Israel |
| **Continued from previous page** | | | | | | | | | | | | | |
| **BMI Group** |  |  |  |  |  |  |  |  |  |  |  |  |  |
| BMI (mean, kg/m^2^) | 27.0 | 26.3 | 28.1 | 26.1 | 26.6 | 27.3 | 25.9 | 26.5 | 27.8 | 27.0 | 27.7 | 27.2 | 27.8 |
| Normal (<25) | 36.8 | 43.2 | 31.1 | 45.2 | 38.1 | 34.9 | 45.5 | 42.6 | 27.9 | 35.7 | 27.7 | 30.3 | 27.6 |
| Overweight (25-30) | 40.8 | 38.3 | 37.6 | 38.8 | 41.9 | 41.5 | 38.8 | 35.4 | 44.4 | 42.7 | 47.2 | 49.3 | 46.1 |
| Obese (>30) | 22.4 | 18.5 | 31.3 | 16.0 | 20.0 | 23.6 | 15.7 | 22.0 | 27.7 | 21.7 | 25.1 | 20.4 | 26.3 |
| **Health Condition*** | | |  |  |  |  |  |  |  |  |  |  |  |
| Hypertension | 42.3 | 40.0 | 53.8 | 34.4 | 34.1 | 43.2 | 31.9 | 37.9 | 47.9 | 47.9 | 44.4 | 49.4 | 52.1 |
| High Blood Cholesterol | 25.5 | 16.9 | 22.5 | 25.1 | 32.3 | 19.9 | 17.7 | 23.8 | 28.5 | 29.8 | 33.9 | 44.5 | 44.9 |
| Stroke | 3.3 | 3.7 | 4.6 | 3.2 | 3.4 | 2.7 | 1.7 | 2.3 | 4.2 | 2.3 | 2.2 | 2.9 | 4.4 |
| Mental Health Problem ** | 1.2 | 1.1 | 1.5 | 0.6 | 1.0 | 1.0 | 0.6 | 0.3 | 1.9 | 1.3 | 1.8 | 2.0 | 3.0 |
| Chronic Kidney Disease | 2.0 | 0.7 | 4.1 | 1.1 | 1.6 | 2.0 | 0.8 | 1.9 | 2.2 | 2.0 | 1.6 | 1.2 | 3.9 |
| Depression | 40.6 | 32.7 | 48.0 | 31.2 | 41.3 | 47.4 | 38.8 | 45.2 | 40.1 | 43.4 | 39.8 | 33.0 | 42.4 |
| Sleep Problem | 37.9 | 32.5 | 51.2 | 31.5 | 35.1 | 38.0 | 33.4 | 40.0 | 41.2 | 34.7 | 34.7 | 30.7 | 46.1 |
| **Healthcare Usage** |  |  |  |  |  |  |  |  |  |  |  |  |  |
| Doctor Visit Last Year | 90.2 | 86.7 | 89.0 | 87.7 | 94.4 | 95.1 | 89.0 | 94.4 | 88.6 | 89.7 | 92.1 | 81.4 | 94.7 |
| Forgo care - cost | 2.9 | 0.2 | 3.6 | 0.2 | 2.5 | 2.4 | 0.8 | 2.6 | 2.1 | 10.4 | 1.6 | 19.4 | 4.4 |
| Forgo care - long wait | 9.5 | 5.9 | 24.0 | 4.1 | 4.7 | 4.6 | 0.9 | 4.6 | 5.8 | 23.5 | 4.7 | 21.6 | 12.0 |
| *: Have you ever been diagnosed with any of the following health conditions?  **: Alzheimer's disease, dementia, senility  Source: The Survey of Health, Ageing, and Retirement in Europe, Wave 6 in 2015 | | | | | | | | | | | | | |

eTable 5：Descriptive statistics of the study sample by diabetes classification

| **Baseline Characteristic** | **Total** | | **Group 1** | | **Group 2** | | **Group 3** | | **Group 4** | |
| --- | --- | --- | --- | --- | --- | --- | --- | --- | --- | --- |
|  |  |  | **normoglycemia** | | **preDM** | | **uDM** | | **dDM** | |
|  | **N** | **%** | **N** | **%** | **N** | **%** | **N** | **%** | **N** | **%** |
| N | 19133 |  | 4558 |  | 10510 |  | 1338 |  | 2727 |  |
| **Gender** |  |  |  |  |  |  |  |  |  |  |
| Female | 10931 | 57.1 | 2633 | 57.8 | 6149 | 58.5 | 768 | 57.4 | 1381 | 50.6 |
| Male | 8202 | 42.9 | 1925 | 42.2 | 4361 | 41.5 | 570 | 42.6 | 1346 | 49.4 |
| **Age Group** |  |  |  |  |  |  |  |  |  |  |
| Age (mean, years) | 67.8 |  | 65.8 |  | 68.0 |  | 68.4 |  | 70.2 |  |
| 50-59 | 4236 | 22.1 | 1333 | 29.2 | 2264 | 21.5 | 280 | 20.9 | 359 | 13.2 |
| 60-69 | 7069 | 36.9 | 1724 | 37.8 | 3890 | 37.0 | 478 | 35.7 | 977 | 35.8 |
| 70-79 | 5378 | 28.1 | 1069 | 23.5 | 2970 | 28.3 | 392 | 29.3 | 947 | 34.7 |
| >=80 | 2450 | 12.8 | 432 | 9.5 | 1386 | 13.2 | 188 | 14.1 | 444 | 16.3 |
| **Immigration** |  |  |  |  |  |  |  |  |  |  |
| No | 16975 | 88.7 | 4079 | 89.5 | 9406 | 89.5 | 1192 | 89.1 | 2298 | 84.3 |
| Yes | 2158 | 11.3 | 479 | 10.5 | 1104 | 10.5 | 146 | 10.9 | 429 | 15.7 |
| **Cohabitation** |  |  |  |  |  |  |  |  |  |  |
| No | 6180 | 32.3 | 1421 | 31.2 | 3391 | 32.3 | 411 | 30.7 | 957 | 35.1 |
| Yes | 12953 | 67.7 | 3137 | 68.8 | 7119 | 67.7 | 927 | 69.3 | 1770 | 64.9 |
| **ISCED Group** |  |  |  |  |  |  |  |  |  |  |
| Years of Education (mean, years) | 11.4 |  | 12.0 |  | 11.4 |  | 10.8 |  | 10.5 |  |
| Low Edu | 6279 | 32.8 | 1233 | 27.1 | 3354 | 31.9 | 535 | 40.0 | 1157 | 42.4 |
| Medium Edu | 7711 | 40.3 | 1829 | 40.1 | 4308 | 41.0 | 515 | 38.5 | 1059 | 38.8 |
| High Edu | 5143 | 26.9 | 1496 | 32.8 | 2848 | 27.1 | 288 | 21.5 | 511 | 18.7 |
| **Income Quartiles** |  |  |  |  |  |  |  |  |  |  |
| Household Income (mean, euro) | 35991 |  | 40994 |  | 36118 |  | 32464 |  | 28939 |  |
| 1st quantiles | 4538 | 23.7 | 980 | 21.5 | 2417 | 23.0 | 355 | 26.5 | 786 | 28.8 |
| 2nd quantiles | 5007 | 26.2 | 1088 | 23.9 | 2779 | 26.4 | 348 | 26.0 | 792 | 29.0 |
| 3rd quantiles | 4959 | 25.9 | 1193 | 26.2 | 2774 | 26.4 | 333 | 24.9 | 659 | 24.2 |
| 4th quantiles | 4629 | 24.2 | 1297 | 28.5 | 2540 | 24.2 | 302 | 22.6 | 490 | 18.0 |
| **Physical Inactivity** |  |  |  |  |  |  |  |  |  |  |
| No | 17556 | 91.8 | 4280 | 93.9 | 9754 | 92.8 | 1220 | 91.2 | 2302 | 84.4 |
| Yes | 1577 | 8.2 | 278 | 6.1 | 756 | 7.2 | 118 | 8.8 | 425 | 15.6 |
| **Ever Smoked** |  |  |  |  |  |  |  |  |  |  |
| No | 10102 | 52.8 | 2404 | 52.7 | 5582 | 53.1 | 706 | 52.8 | 1410 | 51.7 |
| Yes | 9031 | 47.2 | 2154 | 47.3 | 4928 | 46.9 | 632 | 47.2 | 1317 | 48.3 |
| **Alcohol** |  |  |  |  |  |  |  |  |  |  |
| Non-drinkers | 14983 | 78.3 | 3385 | 74.3 | 8260 | 78.6 | 1078 | 80.6 | 2260 | 82.9 |
| Moderate drinkers | 3033 | 15.9 | 865 | 19.0 | 1641 | 15.6 | 180 | 13.5 | 347 | 12.7 |
| Frequent drinkers | 1117 | 5.8 | 308 | 6.8 | 609 | 5.8 | 80 | 6.0 | 120 | 4.4 |
| **Self-reported Health** |  |  |  |  |  |  |  |  |  |  |
| Very good | 5024 | 26.3 | 1591 | 34.9 | 2853 | 27.1 | 341 | 25.5 | 239 | 8.8 |
| Good | 6765 | 35.4 | 1544 | 33.9 | 3879 | 36.9 | 492 | 36.8 | 850 | 31.2 |
| Fair/Poor | 7344 | 38.4 | 1423 | 31.2 | 3778 | 35.9 | 505 | 37.7 | 1638 | 60.1 |
| **BMI Group** |  |  |  |  |  |  |  |  |  |  |
| BMI (mean, kg/m^2^) | 27.0 |  | 25.9 |  | 26.8 |  | 27.5 |  | 29.5 |  |
| Normal (<25) | 7024 | 36.7 | 2073 | 45.5 | 4019 | 38.2 | 415 | 31.0 | 517 | 19.0 |
| Overweight (25-30) | 7816 | 40.9 | 1823 | 40.0 | 4320 | 41.1 | 585 | 43.7 | 1088 | 39.9 |
| Obese (>30) | 4293 | 22.4 | 662 | 14.5 | 2171 | 20.7 | 338 | 25.3 | 1122 | 41.1 |
| **Hypertension** |  |  |  |  |  |  |  |  |  |  |
| No | 11044 | 57.7 | 3060 | 67.1 | 6320 | 60.1 | 739 | 55.2 | 925 | 33.9 |
| Yes | 8089 | 42.3 | 1498 | 32.9 | 4190 | 39.9 | 599 | 44.8 | 1802 | 66.1 |
| **High Blood Cholesterol** |  |  |  |  |  |  |  |  |  |  |
| No | 14270 | 74.6 | 3664 | 80.4 | 8064 | 76.7 | 1016 | 75.9 | 1526 | 56.0 |
| Yes | 4863 | 25.4 | 894 | 19.6 | 2446 | 23.3 | 322 | 24.1 | 1201 | 44.0 |
| **Stroke** |  |  |  |  |  |  |  |  |  |  |
| No | 18420 | 96.3 | 4425 | 97.1 | 10162 | 96.7 | 1296 | 96.9 | 2537 | 93.0 |
| Yes | 713 | 3.7 | 133 | 2.9 | 348 | 3.3 | 42 | 3.1 | 190 | 7.0 |
| **Mental Health Problem *** |  |  |  |  |  |  |  |  |  |  |
| No | 18918 | 98.9 | 4513 | 99.0 | 10402 | 99.0 | 1327 | 99.2 | 2676 | 98.1 |
| Yes | 215 | 1.1 | 45 | 1.0 | 108 | 1.0 | 11 | 0.8 | 51 | 1.9 |
| **Chronic Kidney Disease** |  |  |  |  |  |  |  |  |  |  |
| No | 18761 | 98.1 | 4492 | 98.6 | 10338 | 98.4 | 1312 | 98.1 | 2619 | 96.0 |
| Yes | 372 | 1.9 | 66 | 1.4 | 172 | 1.6 | 26 | 1.9 | 108 | 4.0 |
| **Depression** |  |  |  |  |  |  |  |  |  |  |
| No | 11384 | 59.5 | 2719 | 59.7 | 6293 | 59.9 | 820 | 61.3 | 1552 | 56.9 |
| Yes | 7749 | 40.5 | 1839 | 40.3 | 4217 | 40.1 | 518 | 38.7 | 1175 | 43.1 |
| **Sleep Problem** |  |  |  |  |  |  |  |  |  |  |
| No | 11910 | 62.2 | 2909 | 63.8 | 6556 | 62.4 | 889 | 66.4 | 1556 | 57.1 |
| Yes | 7223 | 37.8 | 1649 | 36.2 | 3954 | 37.6 | 449 | 33.6 | 1171 | 42.9 |
| **Doctor Visit Last Year** |  |  |  |  |  |  |  |  |  |  |
| No | 1866 | 9.8 | 527 | 11.6 | 1118 | 10.6 | 155 | 11.6 | 66 | 2.4 |
| Yes | 17267 | 90.2 | 4031 | 88.4 | 9392 | 89.4 | 1183 | 88.4 | 2661 | 97.6 |
| **Forgo care - cost** |  |  |  |  |  |  |  |  |  |  |
| No | 18578 | 97.1 | 4462 | 97.9 | 10221 | 97.3 | 1290 | 96.4 | 2605 | 95.5 |
| Yes | 555 | 2.9 | 96 | 2.1 | 289 | 2.7 | 48 | 3.6 | 122 | 4.5 |
| **Forgo care – long wait** |  |  |  |  |  |  |  |  |  |  |
| No | 17318 | 90.5 | 4189 | 91.9 | 9529 | 90.7 | 1236 | 92.4 | 2364 | 86.7 |
| Yes | 1815 | 9.5 | 369 | 8.1 | 981 | 9.3 | 102 | 7.6 | 363 | 13.3 |
| Source: The survey of Health, Ageing, and Retirement in Europe, Wave 6 in 2015, 11 European countries and Israel  Note: PreDM: prediabetes mellitus, uDM: undiagnosed diabetes mellitus, dDM: diagnosed diabetes mellitus. The 12 countries studied include Sweden, Estonia, Denmark, Belgium, Germany, Switzerland, France, Slovenia, Italy, Spain, Greece, and Israel.  *: Alzheimer's disease, dementia, senility | | | | | | | | | | |

The characteristics of the four diabetes groups are presented in Supplementary eTable 5. Group 4 (the diagnosed, dDM) has the highest average age (70.2 years), lowest level of education (10.5 years), lowest annual household income (€28,939), highest average body mass index (BMI; 29.5 kg/m^2^), and the highest proportion of hypertensive patients (66.1%), yet the lowest alcohol consumption. Group 3 (the undiagnosed, uDM) is slightly younger (68.4 years), slightly higher educated (10.8 years) with higher annual household income (€32,464) and lower BMI (27.55 kg/m^2^). In group 2 (preDM), the average age is 68.0 years, education level 11.4 years, annual household income €36,118, and BMI 26.85 kg/m^2^. Compared to group 1 (normoglycemics), more individuals in groups 2-4 are physically inactive. Smoking rates are similar across all groups, around 47%. For high blood cholesterol, stroke, mental health problems, and declining kidney function, the prevalence is similar between the preDM and uDM groups compared to the normoglycemics, but much higher in the dDM group.

eTable 6: Risk factors associated with undiagnosed diabetes vs. normoglycemia: Subsample analysis by BMI group

|  | **Variables** | **Normal BMI** | | **Overweight** | | **Obesity** | |
| --- | --- | --- | --- | --- | --- | --- | --- |
|  |  | undiagnosed  vs  normoglycemic | undiagnosed  vs  normoglycemic | undiagnosed  vs  normoglycemic | undiagnosed  vs  normoglycemic | undiagnosed  vs  normoglycemic | undiagnosed  vs  normoglycemic |
| ***Socio- Demographics*** | Male (ref: Female) | 0.72 | 0.76 | 0.89 | 0.86 | 1.64 | 1.76* |
|  |  | [0.44 - 1.18] | [0.46 - 1.26] | [0.58 - 1.37] | [0.54 - 1.35] | [0.81 - 3.33] | [0.91 - 3.43] |
|  | Age 60–69 (ref: 50–59) | 1.15 | 1.17 | 1.36 | 1.21 | 0.53 | 0.57 |
|  |  | [0.65 - 2.01] | [0.66 - 2.07] | [0.76 - 2.46] | [0.71 - 2.06] | [0.24 - 1.17] | [0.27 - 1.17] |
|  | Age 70-79 | 1.43 | 1.43 | 1.34 | 1.06 | 0.81 | 0.66 |
|  |  | [0.73 - 2.81] | [0.70 - 2.90] | [0.76 - 2.38] | [0.59 - 1.91] | [0.33 - 2.01] | [0.27 - 1.61] |
|  | Age 80+ | 2.05* | 1.96 | 1.09 | 0.82 | 1.41 | 1.37 |
|  |  | [0.94 - 4.44] | [0.83 - 4.63] | [0.51 - 2.36] | [0.38 - 1.77] | [0.45 - 4.40] | [0.46 - 4.08] |
|  | Immigrant (ref: born native) | 0.66 | 0.54 | 0.74 | 0.82 | 1.13 | 1.06 |
|  |  | [0.23 - 1.85] | [0.22 - 1.32] | [0.31 - 1.78] | [0.35 - 1.92] | [0.32 - 3.98] | [0.36 - 3.12] |
|  | Cohabiting (ref: non-cohabiting) | 1.74* | 1.81** | 0.86 | 0.88 | 0.89 | 1.13 |
|  |  | [1.00 - 3.02] | [1.03 - 3.20] | [0.52 - 1.41] | [0.53 - 1.45] | [0.43 - 1.83] | [0.52 - 2.47] |
| ***Socio-economic Status*** | Medium Edu (ref: Low Edu) | 0.52* | 0.52* | 1.45 | 1.47 | 1.26 | 1.06 |
|  |  | [0.26 - 1.05] | [0.26 - 1.04] | [0.78 - 2.68] | [0.81 - 2.66] | [0.46 - 3.46] | [0.49 - 2.29] |
|  | High Edu | 0.46** | 0.45** | 0.60 | 0.62 | 1.30 | 0.89 |
|  |  | [0.23 - 0.92] | [0.21 - 0.96] | [0.27 - 1.32] | [0.29 - 1.34] | [0.49 - 3.48] | [0.36 - 2.20] |
|  | Income 2nd quantiles (ref: 1st) | 1.60 | 1.88 | 1.31 | 1.24 | 0.43* | 0.49 |
|  |  | [0.77 - 3.33] | [0.88 - 4.03] | [0.68 - 2.51] | [0.66 - 2.34] | [0.16 - 1.14] | [0.20 - 1.20] |
|  | Income 3rd quantiles | 1.40 | 1.63 | 1.16 | 1.00 | 0.56 | 0.61 |
|  |  | [0.71 - 2.75] | [0.78 - 3.41] | [0.58 - 2.33] | [0.51 - 1.96] | [0.23 - 1.37] | [0.26 - 1.44] |
|  | Income 4th quantiles | 0.73 | 0.93 | 1.02 | 0.94 | 0.21*** | 0.26*** |
|  |  | [0.34 - 1.56] | [0.42 - 2.03] | [0.50 - 2.10] | [0.47 - 1.88] | [0.07 - 0.67] | [0.11 - 0.65] |
| ***Health Behavior*** | Physical Inactivity |  | 1.34 |  | 1.05 |  | 0.45 |
|  |  |  | [0.56 - 3.22] |  | [0.48 - 2.30] |  | [0.17 - 1.19] |
|  | Ever Smoked |  | 1.14 |  | 0.82 |  | 2.27*** |
|  |  |  | [0.70 - 1.84] |  | [0.53 - 1.28] |  | [1.24 - 4.17] |
|  | Alcohol Use: Moderate (ref: No) |  | 0.59 |  | 0.48* |  | 1.04 |
|  |  |  | [0.30 - 1.15] |  | [0.22 - 1.02] |  | [0.39 - 2.80] |
|  | Alcohol Use: Frequent |  | 0.78 |  | 0.71 |  | 0.28** |
|  |  |  | [0.35 - 1.76] |  | [0.32 - 1.58] |  | [0.10 - 0.78] |
| ***Health Condition*** | Self-rated Health: Good (ref: Very good/excellent) |  | 1.00 |  | 0.70 |  | 1.11 |
|  |  |  | [0.57 - 1.74] |  | [0.42 - 1.18] |  | [0.47 - 2.64] |
|  | Self-rated Health: Fair/Poor |  | 1.10 |  | 0.84 |  | 1.72 |
|  |  |  | [0.57 - 2.11] |  | [0.45 - 1.57] |  | [0.67 - 4.39] |
|  | Hypertension |  | 1.61* |  | 1.58* |  | 2.17** |
|  |  |  | [0.99 - 2.62] |  | [0.99 - 2.53] |  | [1.18 - 4.02] |
|  | High Blood Cholesterol |  | 0.69 |  | 0.71 |  | 2.20** |
|  |  |  | [0.39 - 1.23] |  | [0.44 - 1.13] |  | [1.10 - 4.40] |
|  | Stroke |  | 0.92 |  | 5.39*** |  | 0.39 |
|  |  |  | [0.12 - 6.90] |  | [1.73 - 16.80] |  | [0.06 - 2.45] |
|  | Mental Health |  | 0.80 |  | 2.26 |  | 0.39 |
|  |  |  | [0.15 - 4.27] |  | [0.51 - 10.07] |  | [0.05 - 3.22] |
|  | Chronic Kidney Disease |  | 2.50 |  | 1.27 |  | 0.20 |
|  |  |  | [0.41 - 15.32] |  | [0.37 - 4.33] |  | [0.03 - 1.50] |
|  | Depression |  | 1.12 |  | 0.67 |  | 1.97** |
|  |  |  | [0.70 - 1.81] |  | [0.42 - 1.08] |  | [1.07 - 3.63] |
|  | Sleep Problem |  | 0.89 |  | 0.85 |  | 0.94 |
|  |  |  | [0.56 - 1.40] |  | [0.47 - 1.51] |  | [0.45 - 1.97] |
| ***Healthcare Usage*** | Doctor Visit Last Year |  | 1.28 |  | 1.09 |  | 0.77 |
|  |  |  | [0.46 - 3.53] |  | [0.55 - 2.15] |  | [0.27 - 2.19] |
|  | Forgo care - cost |  | 3.31* |  | 1.11 |  | 0.35 |
|  |  |  | [0.86 - 12.70] |  | [0.50 - 2.50] |  | [0.08 - 1.55] |
|  | Forgo care - long wait |  | 0.63 |  | 0.48** |  | 1.24 |
|  |  |  | [0.29 - 1.38] |  | [0.25 - 0.95] |  | [0.48 - 3.18] |
| **Country** | Estonia (baseline: Sweden) | 1.20 | 1.16 | 0.94 | 1.15 | 0.48* | 0.38* |
|  |  | [0.68 - 2.11] | [0.60 - 2.24] | [0.56 - 1.60] | [0.62 - 2.12] | [0.21 - 1.10] | [0.14 - 1.02] |
|  | Denmark | 0.74 | 0.79 | 0.55** | 0.60* | 0.29*** | 0.29** |
|  |  | [0.43 - 1.27] | [0.44 - 1.40] | [0.33 - 0.91] | [0.34 - 1.06] | [0.12 - 0.71] | [0.10 - 0.85] |
|  | **To be continued on next page** | | | | | | |

| **Continued from previous page** | | | | | | | |
| --- | --- | --- | --- | --- | --- | --- | --- |
|  | **Variables** | **Normal BMI** | | **Overweight** | | **Obesity** | |
| **Country** | Belgium | 0.79 | 0.80 | 1.05 | 1.31 | 0.58 | 0.56 |
|  |  | [0.43 - 1.44] | [0.42 - 1.53] | [0.63 - 1.73] | [0.75 - 2.30] | [0.25 - 1.33] | [0.21 - 1.47] |
|  | Germany | 2.13*** | 2.09** | 1.32 | 1.51 | 1.12 | 0.99 |
|  |  | [1.24 - 3.65] | [1.19 - 3.68] | [0.78 - 2.22] | [0.85 - 2.68] | [0.48 - 2.63] | [0.38 - 2.58] |
|  | Switzerland | 0.82 | 0.81 | 0.76 | 0.81 | 0.98 | 0.92 |
|  |  | [0.44 - 1.53] | [0.42 - 1.55] | [0.41 - 1.38] | [0.42 - 1.55] | [0.39 - 2.42] | [0.30 - 2.79] |
|  | France | 0.79 | 0.74 | 1.55 | 1.84 | 0.89 | 0.64 |
|  |  | [0.33 - 1.91] | [0.30 - 1.85] | [0.62 - 3.89] | [0.75 - 4.54] | [0.25 - 3.19] | [0.20 - 2.02] |
|  | Slovenia | 1.98** | 1.95* | 1.88** | 2.01** | 1.51 | 1.34 |
|  |  | [1.06 - 3.72] | [0.99 - 3.85] | [1.04 - 3.41] | [1.04 - 3.88] | [0.60 - 3.79] | [0.45 - 3.98] |
|  | Italy | 1.63 | 1.25 | 1.62 | 1.92* | 1.31 | 1.11 |
|  |  | [0.84 - 3.15] | [0.59 - 2.66] | [0.86 - 3.04] | [0.96 - 3.82] | [0.45 - 3.85] | [0.35 - 3.49] |
|  | Spain | 4.87*** | 4.91*** | 2.60** | 2.91*** | 2.04 | 1.63 |
|  |  | [2.08 - 11.42] | [2.04 - 11.84] | [1.21 - 5.59] | [1.36 - 6.21] | [0.51 - 8.19] | [0.47 - 5.67] |
|  | Greece | 4.28*** | 4.74*** | 4.34*** | 6.19*** | 5.82** | 5.26** |
|  |  | [2.02 - 9.07] | [1.95 - 11.55] | [2.13 - 8.82] | [2.71 - 14.15] | [1.49 - 22.68] | [1.35 - 20.46] |
|  | Israel | 3.29* | 3.42* | 0.54 | 0.58 | 1.18 | 1.40 |
|  |  | [0.88 - 12.39] | [0.93 - 12.52] | [0.21 - 1.38] | [0.22 - 1.53] | [0.25 - 5.64] | [0.20 - 9.78] |
|  | **Observations** | 2,488 | 2,488 | 2,408 | 2,408 | 1000 | 1000 |

Exponentiated coefficients * p<0.10, ** p<0.05, *** p<0.01

Source: The Survey of Health, Ageing, and Retirement in Europe, Wave 6 in 2015

eTable 7: Risk factors associated with undiagnosed diabetes vs. prediabetes: Subsample analysis by BMI group

|  | **Variables** | **Normal BMI** | | **Overweight** | | **Obesity** | |
| --- | --- | --- | --- | --- | --- | --- | --- |
|  |  | undiagnosed  vs  prediabetic | undiagnosed vs  prediabetic | undiagnosed  vs  prediabetic | undiagnosed vs  prediabetic | undiagnosed vs  prediabetic | undiagnosed vs  prediabetic |
| ***Socio- Demographics*** | Male (ref: Female) | 0.93 | 0.90 | 1.06 | 1.14 | 1.41 | 1.37 |
|  |  | [0.60 - 1.46] | [0.58 - 1.40] | [0.71 - 1.59] | [0.73 - 1.79] | [0.83 - 2.40] | [0.81 - 2.32] |
|  | Age 60–69 (ref: 50–59) | 0.88 | 0.97 | 0.99 | 0.88 | 0.51** | 0.57** |
|  |  | [0.52 - 1.49] | [0.58 - 1.63] | [0.59 - 1.66] | [0.53 - 1.44] | [0.27 - 0.96] | [0.33 - 0.98] |
|  | Age 70-79 | 0.84 | 0.90 | 0.79 | 0.64* | 0.65 | 0.72 |
|  |  | [0.45 - 1.57] | [0.48 - 1.67] | [0.49 - 1.28] | [0.38 - 1.08] | [0.32 - 1.31] | [0.38 - 1.36] |
|  | Age 80+ | 1.16 | 1.28 | 0.61 | 0.41** | 0.63 | 0.84 |
|  |  | [0.56 - 2.37] | [0.63 - 2.60] | [0.31 - 1.18] | [0.19 - 0.88] | [0.23 - 1.70] | [0.34 - 2.08] |
|  | Immigrant (ref: born native) | 0.66 | 0.59 | 1.24 | 1.25 | 0.84 | 0.95 |
|  |  | [0.25 - 1.79] | [0.26 - 1.34] | [0.60 - 2.56] | [0.62 - 2.50] | [0.33 - 2.17] | [0.39 - 2.28] |
|  | Cohabiting (ref: non-cohabiting) | 1.37 | 1.43 | 0.87 | 0.87 | 0.63 | 0.63 |
|  |  | [0.79 - 2.37] | [0.82 - 2.47] | [0.55 - 1.38] | [0.56 - 1.36] | [0.36 - 1.11] | [0.36 - 1.12] |
| ***Socio-economic Status*** | Medium Edu (ref: Low Edu) | 0.59 | 0.64 | 1.13 | 1.18 | 1.15 | 0.97 |
|  |  | [0.32 - 1.11] | [0.35 - 1.16] | [0.68 - 1.88] | [0.72 - 1.93] | [0.51 - 2.57] | [0.51 - 1.85] |
|  | High Edu | 0.58* | 0.62 | 0.60 | 0.60 | 0.64 | 0.62 |
|  |  | [0.31 - 1.10] | [0.33 - 1.14] | [0.29 - 1.26] | [0.29 - 1.22] | [0.29 - 1.44] | [0.30 - 1.27] |
|  | Income 2nd quantiles (ref: 1st) | 1.14 | 1.14 | 1.01 | 0.98 | 0.65 | 0.76 |
|  |  | [0.60 - 2.15] | [0.61 - 2.15] | [0.57 - 1.81] | [0.56 - 1.72] | [0.33 - 1.25] | [0.42 - 1.36] |
|  | Income 3rd quantiles | 1.18 | 1.16 | 1.09 | 1.07 | 0.85 | 0.89 |
|  |  | [0.63 - 2.22] | [0.61 - 2.19] | [0.61 - 1.94] | [0.60 - 1.89] | [0.41 - 1.77] | [0.45 - 1.77] |
|  | Income 4th quantiles | 0.64 | 0.64 | 1.10 | 1.09 | 0.77 | 0.96 |
|  |  | [0.31 - 1.30] | [0.32 - 1.28] | [0.60 - 2.02] | [0.60 - 2.00] | [0.33 - 1.80] | [0.43 - 2.14] |
| ***Health Behavior*** | Physical Inactivity |  | 1.13 |  | 1.35 |  | 0.60 |
|  |  |  | [0.52 - 2.48] |  | [0.73 - 2.49] |  | [0.29 - 1.24] |
|  | Ever Smoked |  | 1.13 |  | 0.80 |  | 1.33 |
|  |  |  | [0.74 - 1.72] |  | [0.53 - 1.22] |  | [0.78 - 2.25] |
|  | Alcohol Use: Moderate (ref: No) |  | 0.65 |  | 0.51** |  | 1.64 |
|  |  |  | [0.34 - 1.25] |  | [0.28 - 0.94] |  | [0.79 - 3.39] |
|  | Alcohol Use: Frequent |  | 0.74 |  | 0.68 |  | 1.21 |
|  |  |  | [0.35 - 1.57] |  | [0.31 - 1.52] |  | [0.50 - 2.90] |
| ***Health Condition*** | Self-rated Health: Good (ref: Very good/excellent) |  | 0.70 |  | 0.90 |  | 0.86 |
|  |  |  | [0.42 - 1.17] |  | [0.55 - 1.46] |  | [0.46 - 1.60] |
|  | Self-rated Health: Fair/Poor |  | 0.85 |  | 1.26 |  | 1.41 |
|  |  |  | [0.47 - 1.55] |  | [0.74 - 2.13] |  | [0.71 - 2.81] |
|  | Hypertension |  | 1.16 |  | 1.54* |  | 1.88** |
|  |  |  | [0.76 - 1.78] |  | [1.00 - 2.38] |  | [1.15 - 3.07] |
|  | High Blood Cholesterol |  | 0.67* |  | 0.59** |  | 0.91 |
|  |  |  | [0.42 - 1.07] |  | [0.38 - 0.94] |  | [0.53 - 1.55] |
|  | Stroke |  | 0.94 |  | 2.30 |  | 0.12*** |
|  |  |  | [0.14 - 6.20] |  | [0.80 - 6.61] |  | [0.03 - 0.44] |
|  | Mental Health |  | 0.85 |  | 0.24* |  | 0.85 |
|  |  |  | [0.21 - 3.44] |  | [0.05 - 1.12] |  | [0.14 - 5.24] |
|  | Chronic Kidney Disease |  | 1.88 |  | 1.21 |  | 0.16** |
|  |  |  | [0.40 - 8.76] |  | [0.46 - 3.19] |  | [0.03 - 0.92] |
|  | Depression |  | 1.00 |  | 0.73 |  | 1.30 |
|  |  |  | [0.66 - 1.53] |  | [0.48 - 1.11] |  | [0.80 - 2.11] |
|  | Sleep Problem |  | 0.71* |  | 0.90 |  | 1.03 |
|  |  |  | [0.48 - 1.04] |  | [0.57 - 1.43] |  | [0.61 - 1.74] |
| ***Healthcare Usage*** | Doctor Visit Last Year |  | 1.17 |  | 0.77 |  | 0.83 |
|  |  |  | [0.52 - 2.61] |  | [0.43 - 1.37] |  | [0.38 - 1.84] |
|  | Forgo care - cost |  | 1.51 |  | 0.90 |  | 0.44 |
|  |  |  | [0.60 - 3.81] |  | [0.43 - 1.90] |  | [0.15 - 1.30] |
|  | Forgo care - long wait |  | 1.37 |  | 0.43*** |  | 1.90 |
|  |  |  | [0.70 - 2.67] |  | [0.25 - 0.77] |  | [0.87 - 4.14] |
| **Country** | Estonia (baseline: Sweden) | 1.27 | 1.32 | 0.74 | 0.72 | 0.34*** | 0.23*** |
|  |  | [0.76 - 2.12] | [0.74 - 2.36] | [0.46 - 1.19] | [0.42 - 1.22] | [0.18 - 0.63] | [0.11 - 0.47] |
|  | Denmark | 1.25 | 1.26 | 0.87 | 1.02 | 0.34*** | 0.29*** |
|  |  | [0.75 - 2.07] | [0.75 - 2.11] | [0.54 - 1.41] | [0.61 - 1.71] | [0.16 - 0.70] | [0.13 - 0.65] |
|  | Belgium | 1.04 | 1.11 | 1.14 | 1.28 | 0.49** | 0.56 |
|  |  | [0.60 - 1.82] | [0.62 - 1.99] | [0.73 - 1.80] | [0.78 - 2.08] | [0.25 - 0.95] | [0.27 - 1.16] |
| **To be continued on next page** | | | | | | | |

| **Continued from previous page** | | | | | | | |
| --- | --- | --- | --- | --- | --- | --- | --- |
|  | **Variables** | **Normal BMI** | | **Overweight** | | **Obesity** | |
| **Country** | Germany | 2.58*** | 2.76*** | 1.22 | 1.25 | 0.65 | 0.61 |
|  |  | [1.57 - 4.23] | [1.64 - 4.63] | [0.76 - 1.95] | [0.75 - 2.09] | [0.34 - 1.22] | [0.31 - 1.19] |
|  | Switzerland | 0.90 | 0.94 | 0.83 | 0.83 | 0.59 | 0.63 |
|  |  | [0.51 - 1.61] | [0.52 - 1.70] | [0.49 - 1.39] | [0.48 - 1.45] | [0.29 - 1.22] | [0.29 - 1.38] |
|  | France | 1.47 | 1.61 | 1.63 | 1.75 | 1.23 | 0.86 |
|  |  | [0.62 - 3.47] | [0.69 - 3.77] | [0.69 - 3.86] | [0.79 - 3.85] | [0.39 - 3.91] | [0.32 - 2.32] |
|  | Slovenia | 1.86** | 2.11** | 1.36 | 1.29 | 0.95 | 0.95 |
|  |  | [1.05 - 3.29] | [1.17 - 3.80] | [0.82 - 2.25] | [0.75 - 2.22] | [0.51 - 1.76] | [0.48 - 1.88] |
|  | Italy | 2.19** | 2.01** | 1.84** | 2.00** | 0.68 | 0.60 |
|  |  | [1.16 - 4.15] | [1.02 - 3.96] | [1.05 - 3.23] | [1.10 - 3.63] | [0.31 - 1.47] | [0.28 - 1.33] |
|  | Spain | 6.33*** | 6.51*** | 1.94** | 1.94** | 0.85 | 0.88 |
|  |  | [3.28 - 12.21] | [3.32 - 12.79] | [1.06 - 3.55] | [1.05 - 3.58] | [0.34 - 2.16] | [0.35 - 2.21] |
|  | Greece | 4.14*** | 3.85*** | 2.24*** | 2.93*** | 1.59 | 1.30 |
|  |  | [2.20 - 7.79] | [1.84 - 8.04] | [1.26 - 3.98] | [1.55 - 5.53] | [0.65 - 3.92] | [0.50 - 3.37] |
|  | Israel | 10.80*** | 11.22*** | 1.51 | 1.66 | 2.15 | 2.74 |
|  |  | [2.93 - 39.86] | [2.84 - 44.29] | [0.63 - 3.59] | [0.68 - 4.09] | [0.53 - 8.74] | [0.59 - 12.67] |
|  | **Observations** | 4,432 | 4,432 | 4,907 | 4,907 | 2,509 | 2,509 |

Exponentiated coefficients * p<0.10, ** p<0.05, *** p<0.01

Source: The Survey of Health, Ageing, and Retirement in Europe, Wave 6 in 2015

eTable 8: Risk factors associated with undiagnosed vs. diagnosed diabetes: Subsample analysis by BMI group

|  | **Variables** | **Normal BMI** | | **Overweight** | | **Obesity** | |
| --- | --- | --- | --- | --- | --- | --- | --- |
|  |  | undiagnosed vs  diagnosed | undiagnosed vs  diagnosed | undiagnosed  vs  diagnosed | undiagnosed  vs  diagnosed | undiagnosed vs  diagnosed | undiagnosed  vs  diagnosed |
| ***Socio- Demographics*** | Male (ref: Female) | 0.30*** | 0.23*** | 0.53*** | 0.60** | 0.92 | 0.79 |
|  |  | [0.17 - 0.50] | [0.13 - 0.41] | [0.35 - 0.80] | [0.38 - 0.95] | [0.53 - 1.58] | [0.45 - 1.38] |
|  | Age 60–69 (ref: 50–59) | 0.65 | 0.76 | 0.55** | 0.67 | 0.31*** | 0.36*** |
|  |  | [0.30 - 1.42] | [0.35 - 1.65] | [0.30 - 0.99] | [0.37 - 1.20] | [0.16 - 0.61] | [0.20 - 0.66] |
|  | Age 70-79 | 0.49* | 0.64 | 0.37*** | 0.48** | 0.24*** | 0.42** |
|  |  | [0.21 - 1.14] | [0.28 - 1.47] | [0.20 - 0.66] | [0.27 - 0.84] | [0.11 - 0.55] | [0.22 - 0.83] |
|  | Age 80+ | 0.48 | 0.78 | 0.35*** | 0.48* | 0.40* | 0.89 |
|  |  | [0.20 - 1.16] | [0.32 - 1.90] | [0.16 - 0.75] | [0.22 - 1.06] | [0.15 - 1.02] | [0.36 - 2.17] |
|  | Immigrant (ref: born native) | 0.28** | 0.30** | 0.53 | 0.59 | 0.53 | 0.53 |
|  |  | [0.09 - 0.86] | [0.10 - 0.90] | [0.24 - 1.18] | [0.25 - 1.39] | [0.18 - 1.58] | [0.18 - 1.51] |
|  | Cohabiting  (ref: non-cohabiting) | 1.99* | 1.95** | 1.06 | 1.05 | 0.65 | 0.73 |
|  |  | [0.99 - 3.99] | [1.01 - 3.79] | [0.64 - 1.75] | [0.62 - 1.77] | [0.35 - 1.20] | [0.40 - 1.35] |
| ***Socio-economic Status*** | Medium Edu (ref: Low Edu) | 0.41** | 0.47** | 1.56 | 1.54 | 1.06 | 1.08 |
|  |  | [0.18 - 0.92] | [0.23 - 0.97] | [0.88 - 2.75] | [0.85 - 2.80] | [0.52 - 2.17] | [0.58 - 2.03] |
|  | High Edu | 0.96 | 0.83 | 1.26 | 1.05 | 0.97 | 1.06 |
|  |  | [0.45 - 2.04] | [0.38 - 1.78] | [0.66 - 2.41] | [0.53 - 2.07] | [0.46 - 2.06] | [0.51 - 2.21] |
|  | Income 2nd quantiles (ref: 1st) | 1.41 | 1.40 | 1.53 | 1.31 | 0.69 | 0.64 |
|  |  | [0.68 - 2.94] | [0.66 - 2.96] | [0.82 - 2.83] | [0.70 - 2.44] | [0.35 - 1.36] | [0.33 - 1.26] |
|  | Income 3rd quantiles | 1.18 | 1.20 | 1.44 | 1.20 | 1.81 | 1.68 |
|  |  | [0.52 - 2.70] | [0.53 - 2.73] | [0.74 - 2.79] | [0.57 - 2.54] | [0.82 - 4.00] | [0.79 - 3.58] |
|  | Income 4th quantiles | 0.96 | 0.58 | 2.05** | 1.91* | 1.11 | 1.14 |
|  |  | [0.35 - 2.66] | [0.22 - 1.52] | [1.03 - 4.08] | [0.95 - 3.86] | [0.47 - 2.59] | [0.50 - 2.59] |
| ***Health Behavior*** | Physical Inactivity |  | 0.95 |  | 0.99 |  | 0.22*** |
|  |  |  | [0.42 - 2.17] |  | [0.50 - 1.94] |  | [0.10 - 0.50] |
|  | Ever Smoked |  | 1.15 |  | 0.73 |  | 1.00 |
|  |  |  | [0.67 - 1.96] |  | [0.47 - 1.16] |  | [0.58 - 1.71] |
|  | Alcohol Use: Moderate (ref: No) |  | 0.51 |  | 0.89 |  | 2.75*** |
|  |  |  | [0.19 - 1.34] |  | [0.48 - 1.65] |  | [1.38 - 5.49] |
|  | Alcohol Use: Frequent |  | 1.23 |  | 1.25 |  | 2.36* |
|  |  |  | [0.28 - 5.47] |  | [0.42 - 3.69] |  | [0.95 - 5.85] |
| ***Health Condition*** | Self-rated Health: Good (ref: Very good/excellent) |  | 0.38** |  | 0.31*** |  | 0.43** |
|  |  |  | [0.16 - 0.89] |  | [0.15 - 0.63] |  | [0.19 - 0.99] |
|  | Self-rated Health: Fair/Poor |  | 0.19*** |  | 0.21*** |  | 0.32** |
|  |  |  | [0.08 - 0.46] |  | [0.10 - 0.44] |  | [0.13 - 0.76] |
|  | Hypertension |  | 0.64 |  | 0.90 |  | 0.94 |
|  |  |  | [0.36 - 1.13] |  | [0.57 - 1.43] |  | [0.56 - 1.60] |
|  | High Blood Cholesterol |  | 0.26*** |  | 0.41*** |  | 0.58* |
|  |  |  | [0.14 - 0.47] |  | [0.26 - 0.64] |  | [0.34 - 1.01] |
|  | Stroke |  | 0.37 |  | 1.57 |  | 0.13*** |
|  |  |  | [0.10 - 1.47] |  | [0.56 - 4.38] |  | [0.03 - 0.55] |
|  | Mental Health |  | 1.28 |  | 0.28 |  | 0.95 |
|  |  |  | [0.24 - 6.93] |  | [0.04 - 1.95] |  | [0.21 - 4.25] |
|  | Chronic Kidney Disease |  | 0.96 |  | 1.83 |  | 0.16** |
|  |  |  | [0.20 - 4.58] |  | [0.54 - 6.22] |  | [0.03 - 0.95] |
|  | Depression |  | 1.17 |  | 1.31 |  | 1.53 |
|  |  |  | [0.64 - 2.11] |  | [0.81 - 2.11] |  | [0.89 - 2.62] |
|  | Sleep Problem |  | 0.70 |  | 0.84 |  | 1.19 |
|  |  |  | [0.38 - 1.28] |  | [0.52 - 1.37] |  | [0.66 - 2.15] |
| **Country** | Estonia (baseline: Sweden) | 0.75 | 1.47 | 1.03 | 2.02* | 0.33*** | 0.42** |
|  |  | [0.35 - 1.63] | [0.55 - 3.93] | [0.55 - 1.95] | [0.95 - 4.29] | [0.17 - 0.61] | [0.20 - 0.87] |
|  | Denmark | 0.83 | 0.82 | 1.42 | 1.61 | 0.55 | 0.48 |
|  |  | [0.38 - 1.81] | [0.31 - 2.14] | [0.74 - 2.72] | [0.75 - 3.43] | [0.26 - 1.18] | [0.19 - 1.16] |
|  | Belgium | 0.39** | 0.55 | 1.26 | 1.72 | 0.55* | 0.67 |
|  |  | [0.17 - 0.89] | [0.21 - 1.44] | [0.69 - 2.30] | [0.86 - 3.43] | [0.28 - 1.08] | [0.32 - 1.42] |
|  | Germany | 1.34 | 1.58 | 0.93 | 1.42 | 0.52** | 0.69 |
|  |  | [0.62 - 2.88] | [0.62 - 4.06] | [0.50 - 1.73] | [0.69 - 2.91] | [0.28 - 0.96] | [0.35 - 1.35] |
| **To be continued on next page** | | | | | | | |

| **Continued from previous page** | | | | | | | |
| --- | --- | --- | --- | --- | --- | --- | --- |
|  | **Variables** | **Normal BMI** | | **Overweight** | | **Obesity** | |
| **Country** | Switzerland | 1.66 | 1.35 | 1.47 | 1.64 | 1.02 | 0.91 |
|  |  | [0.65 - 4.24] | [0.42 - 4.36] | [0.73 - 2.96] | [0.75 - 3.57] | [0.42 - 2.47] | [0.33 - 2.52] |
|  | France | 3.35* | 5.72*** | 2.36* | 4.04** | 0.90 | 1.10 |
|  |  | [0.94 - 11.93] | [1.55 - 21.02] | [0.91 - 6.13] | [1.36 - 12.02] | [0.31 - 2.63] | [0.40 - 3.02] |
|  | Slovenia | 0.76 | 1.13 | 1.02 | 1.68 | 1.37 | 2.32** |
|  |  | [0.32 - 1.83] | [0.40 - 3.21] | [0.53 - 1.98] | [0.78 - 3.62] | [0.70 - 2.69] | [1.07 - 5.02] |
|  | Italy | 0.77 | 0.98 | 1.51 | 2.11* | 0.80 | 1.66 |
|  |  | [0.33 - 1.78] | [0.34 - 2.79] | [0.75 - 3.02] | [0.99 - 4.51] | [0.36 - 1.78] | [0.70 - 3.97] |
|  | Spain | 1.27 | 1.36 | 1.43 | 2.18** | 0.39** | 0.55 |
|  |  | [0.46 - 3.50] | [0.48 - 3.83] | [0.69 - 2.96] | [1.00 - 4.74] | [0.16 - 0.96] | [0.22 - 1.42] |
|  | Greece | 0.79 | 1.67 | 1.16 | 2.18* | 2.09 | 2.43* |
|  |  | [0.31 - 2.02] | [0.53 - 5.25] | [0.54 - 2.51] | [0.91 - 5.20] | [0.78 - 5.62] | [0.87 - 6.75] |
|  | Israel | 2.06 | 3.13 | 0.35** | 0.50 | 0.77 | 1.51 |
|  |  | [0.54 - 7.86] | [0.60 - 16.28] | [0.13 - 0.98] | [0.15 - 1.65] | [0.17 - 3.58] | [0.37 - 6.19] |
|  | **Observations** | 932 | 932 | 1,673 | 1,673 | 1460 | 1460 |

Exponentiated coefficients * p<0.10, ** p<0.05, *** p<0.01

Source: The Survey of Health, Ageing, and Retirement in Europe, Wave 6 in 2015

eTable 9: The undiagnosed diabetes group (uDM) in the study sample before and after raising the diagnostic threshold to 7%.

| **Undiagnosed diabetes** | **Diabetes threshold of 6.5%** | | **Diabetes threshold of 7%** | |
| --- | --- | --- | --- | --- |
|  | **N** | **%** | **N** | **%** |
| **N** | **1338** | **7.0** | **404** | **2.1** |
| **Gender** |  |  |  |  |
| Female | 768 | 57.4 | 229 | 56.7 |
| Male | 570 | 42.6 | 175 | 43.3 |
| **Age Group** |  |  |  |  |
| 50-59 | 281 | 21.0 | 92 | 22.8 |
| 60-69 | 477 | 35.7 | 141 | 34.9 |
| 70-79 | 392 | 29.3 | 122 | 30.2 |
| >=80 | 188 | 14.1 | 49 | 12.1 |
| **Education Group** |  |  |  |  |
| Low Edu | 534 | 39.9 | 148 | 36.6 |
| Medium Edu | 515 | 38.5 | 167 | 41.3 |
| High Edu | 289 | 21.6 | 89 | 22.0 |

eTable 10: Sensitivity Analysis Defining Diabetes as HbA1c ≥ 7%: Factors associated with undiagnosed diabetes mellitus (uDM)

|  | **Variables** | (1) | (2) | (3) | (4) | (5) | (6) |
| --- | --- | --- | --- | --- | --- | --- | --- |
|  |  | undiagnosed  vs normoglycemic | undiagnosed  vs  normoglycemic | undiagnosed vs  prediabetic | undiagnosed vs  prediabetic | undiagnosed vs  diagnosed | undiagnosed vs  diagnosed |
| ***Socio- Demographics*** | Male (ref: Female) | 1.20 | 1.02 | 1.21 | 1.05 | 0.58** | 0.58** |
|  |  | [0.69 - 2.10] | [0.60 - 1.75] | [0.73 - 2.00] | [0.65 - 1.71] | [0.36 - 0.93] | [0.36 - 0.93] |
|  | Age 60–69 (ref: 50–59) | 0.81 | 0.77 | 0.60 | 0.55* | 0.34*** | 0.36*** |
|  |  | [0.45 - 1.47] | [0.44 - 1.33] | [0.33 - 1.11] | [0.30 - 1.01] | [0.18 - 0.65] | [0.20 - 0.66] |
|  | Age 70-79 | 1.27 | 1.11 | 0.79 | 0.77 | 0.31*** | 0.39*** |
|  |  | [0.66 - 2.45] | [0.59 - 2.06] | [0.41 - 1.53] | [0.40 - 1.46] | [0.16 - 0.59] | [0.22 - 0.72] |
|  | Age 80+ | 0.88 | 1.03 | 0.54 | 0.57 | 0.29*** | 0.41** |
|  |  | [0.36 - 2.11] | [0.46 - 2.31] | [0.21 - 1.34] | [0.24 - 1.35] | [0.13 - 0.63] | [0.20 - 0.82] |
|  | Immigrant (ref: born native) | 0.72 | 0.82 | 0.85 | 0.97 | 0.52 | 0.53 |
|  |  | [0.29 - 1.77] | [0.38 - 1.80] | [0.36 - 2.01] | [0.47 - 1.99] | [0.24 - 1.14] | [0.21 - 1.34] |
|  | Cohabiting (ref: non-cohabiting) | 0.62* | 0.64* | 0.51*** | 0.51*** | 0.76 | 0.81 |
|  |  | [0.37 - 1.04] | [0.38 - 1.07] | [0.31 - 0.84] | [0.31 - 0.83] | [0.46 - 1.25] | [0.49 - 1.36] |
| ***Socio-economic Status*** | Medium Edu (ref: Low Edu) | 2.23* | 2.77*** | 1.86 | 2.05** | 2.06** | 1.98** |
|  |  | [0.93 - 5.33] | [1.29 - 5.93] | [0.86 - 4.05] | [1.09 - 3.84] | [1.08 - 3.92] | [1.08 - 3.60] |
|  | High Edu | 1.06 | 1.53 | 1.16 | 1.32 | 2.47*** | 2.02** |
|  |  | [0.44 - 2.56] | [0.68 - 3.45] | [0.51 - 2.64] | [0.65 - 2.71] | [1.26 - 4.85] | [1.04 - 3.93] |
|  | Income 2nd quantiles (ref: 1st) | 1.11 | 1.31 | 0.94 | 1.08 | 1.41 | 1.34 |
|  |  | [0.50 - 2.49] | [0.66 - 2.59] | [0.39 - 2.25] | [0.59 - 1.98] | [0.72 - 2.75] | [0.74 - 2.42] |
|  | Income 3rd quantiles | 0.81 | 0.97 | 0.97 | 1.05 | 1.36 | 1.05 |
|  |  | [0.33 - 1.98] | [0.49 - 1.91] | [0.43 - 2.17] | [0.55 - 2.03] | [0.67 - 2.78] | [0.51 - 2.15] |
|  | Income 4th quantiles | 0.46 | 0.52* | 0.61 | 0.70 | 1.07 | 1.03 |
|  |  | [0.18 - 1.17] | [0.25 - 1.09] | [0.24 - 1.52] | [0.35 - 1.43] | [0.45 - 2.53] | [0.45 - 2.36] |
| ***Health Behavior*** | Physical Inactivity |  | 0.56 |  | 0.56 |  | 0.33*** |
|  |  |  | [0.23 - 1.35] |  | [0.26 - 1.21] |  | [0.15 - 0.71] |
|  | Ever Smoked |  | 0.91 |  | 0.86 |  | 0.70 |
|  |  |  | [0.55 - 1.49] |  | [0.53 - 1.39] |  | [0.43 - 1.15] |
|  | Alcohol Use: Moderate (ref: No) |  | 1.30 |  | 1.47 |  | 2.59** |
|  |  |  | [0.58 - 2.93] |  | [0.71 - 3.05] |  | [1.20 - 5.57] |
|  | Alcohol Use: Frequent |  | 0.94 |  | 1.31 |  | 2.29* |
|  |  |  | [0.39 - 2.27] |  | [0.61 - 2.83] |  | [0.90 - 5.80] |
| ***Health Condition*** | Self-rated Health: Good (ref: Very good/excellent) |  | 0.90 |  | 0.87 |  | 0.40*** |
|  |  |  | [0.52 - 1.54] |  | [0.52 - 1.48] |  | [0.22 - 0.75] |
|  | Self-rated Health: Fair/Poor |  | 1.08 |  | 1.10 |  | 0.23*** |
|  |  |  | [0.56 - 2.10] |  | [0.55 - 2.20] |  | [0.11 - 0.45] |
|  | BMI 25–29.9 (Overweight, ref: <25 Normal) |  | 1.92** |  | 1.30 |  | 0.72 |
|  |  |  | [1.16 - 3.19] |  | [0.82 - 2.05] |  | [0.42 - 1.25] |
|  | BMI ≥30 (Obese) |  | 3.73*** |  | 1.82** |  | 0.50** |
|  |  |  | [2.03 - 6.85] |  | [1.09 - 3.04] |  | [0.26 - 0.94] |
|  | Hypertension |  | 1.64** |  | 1.46 |  | 0.81 |
|  |  |  | [1.00 - 2.70] |  | [0.92 - 2.32] |  | [0.51 - 1.28] |
|  | High Blood Cholesterol |  | 1.41 |  | 1.01 |  | 0.55** |
|  |  |  | [0.84 - 2.36] |  | [0.61 - 1.67] |  | [0.32 - 0.96] |
|  | Stroke |  | 2.98* |  | 2.40 |  | 1.70 |
|  |  |  | [0.96 - 9.31] |  | [0.84 - 6.85] |  | [0.61 - 4.71] |
|  | Mental Health |  | 0.18* |  | 0.08*** |  | 0.11** |
|  |  |  | [0.03 - 1.15] |  | [0.01 - 0.43] |  | [0.02 - 0.65] |
|  | Chronic Kidney Disease |  | 0.80 |  | 0.84 |  | 0.83 |
|  |  |  | [0.17 - 3.81] |  | [0.24 - 2.96] |  | [0.18 - 3.82] |
|  | Depression |  | 1.09 |  | 1.09 |  | 1.46* |
|  |  |  | [0.66 - 1.77] |  | [0.71 - 1.68] |  | [0.93 - 2.29] |
|  | Sleep Problem |  | 0.91 |  | 0.79 |  | 0.74 |
|  |  |  | [0.48 - 1.73] |  | [0.45 - 1.40] |  | [0.44 - 1.26] |
| ***Healthcare Usage*** | Doctor Visit Last Year |  | 0.64 |  | 0.71 |  |  |
|  |  |  | [0.35 - 1.20] |  | [0.38 - 1.33] |  |  |
|  | Forgo care - cost |  | 0.56 |  | 0.41* |  |  |
|  |  |  | [0.19 - 1.68] |  | [0.17 - 1.00] |  |  |
|  | Forgo care - long wait |  | 1.16 |  | 1.73 |  |  |
|  |  |  | [0.52 - 2.56] |  | [0.80 - 3.76] |  |  |
| **To be continued on next page** | | | | | | | |

| **Continued from previous page** | | | | | | | |
| --- | --- | --- | --- | --- | --- | --- | --- |
|  | **Variables** | (1) | (2) | (3) | (4) | (5) | (6) |
|  |  | undiagnosed  vs normoglycemic | undiagnosed  vs  normoglycemic | undiagnosed vs  pre-diabetics | undiagnosed vs  pre-diabetics | undiagnosed vs  diagnosed | undiagnosed vs  diagnosed |
| **Country** | Estonia (baseline: Sweden) | 0.48** | 0.39*** | 0.46*** | 0.35*** | 0.37*** | 0.76 |
|  |  | [0.27 - 0.86] | [0.20 - 0.77] | [0.27 - 0.80] | [0.19 - 0.65] | [0.20 - 0.69] | [0.36 - 1.57] |
|  | Denmark | 0.33*** | 0.30*** | 0.55** | 0.51** | 0.69 | 0.52* |
|  |  | [0.18 - 0.59] | [0.16 - 0.56] | [0.31 - 0.96] | [0.29 - 0.92] | [0.37 - 1.29] | [0.25 - 1.11] |
|  | Belgium | 0.81 | 0.86 | 0.91 | 0.96 | 0.78 | 0.78 |
|  |  | [0.45 - 1.45] | [0.47 - 1.59] | [0.52 - 1.59] | [0.53 - 1.73] | [0.42 - 1.44] | [0.38 - 1.60] |
|  | Germany | 0.75 | 0.77 | 0.75 | 0.72 | 0.52** | 0.66 |
|  |  | [0.42 - 1.33] | [0.43 - 1.39] | [0.44 - 1.28] | [0.41 - 1.27] | [0.29 - 0.94] | [0.32 - 1.32] |
|  | Switzerland | 0.61 | 0.66 | 0.70 | 0.73 | 1.31 | 1.14 |
|  |  | [0.32 - 1.14] | [0.35 - 1.26] | [0.38 - 1.28] | [0.40 - 1.33] | [0.67 - 2.57] | [0.53 - 2.46] |
|  | France | 1.31 | 1.36 | 1.91 | 1.98* | 2.18* | 3.78*** |
|  |  | [0.54 - 3.20] | [0.63 - 2.97] | [0.74 - 4.91] | [0.90 - 4.37] | [0.91 - 5.25] | [1.49 - 9.63] |
|  | Slovenia | 1.70* | 1.58 | 1.35 | 1.29 | 1.05 | 1.62 |
|  |  | [0.94 - 3.08] | [0.81 - 3.07] | [0.79 - 2.30] | [0.74 - 2.27] | [0.58 - 1.92] | [0.78 - 3.38] |
|  | Italy | 1.58 | 2.00* | 1.77* | 1.99* | 1.26 | 1.96* |
|  |  | [0.78 - 3.22] | [0.95 - 4.20] | [0.90 - 3.51] | [0.99 - 3.98] | [0.65 - 2.41] | [0.93 - 4.13] |
|  | Spain | 2.23* | 2.70** | 1.76 | 2.05** | 0.78 | 1.45 |
|  |  | [0.92 - 5.41] | [1.17 - 6.23] | [0.87 - 3.54] | [1.02 - 4.11] | [0.34 - 1.79] | [0.63 - 3.35] |
|  | Greece | 5.10*** | 5.46*** | 2.84*** | 2.89*** | 1.53 | 2.10* |
|  |  | [2.45 - 10.62] | [2.48 - 12.01] | [1.47 - 5.51] | [1.42 - 5.89] | [0.72 - 3.23] | [0.90 - 4.88] |
|  | Israel | 0.90 | 0.90 | 2.33* | 2.44* | 0.52 | 0.89 |
|  |  | [0.34 - 2.41] | [0.33 - 2.45] | [0.89 - 6.10] | [0.93 - 6.36] | [0.21 - 1.33] | [0.29 - 2.72] |
|  | **Observations** | 4,961 | 4,961 | 11,848 | 11,848 | 3,130 | 3,130 |
| **Source**: The Survey of Health, Ageing, and Retirement in Europe, Wave 6 in 2015, 11 European countries and Israel  **Note:** Table presents odds ratios (ORs) and 95% confidence intervals [in brackets] from weighted logistic regression models. All models include country fixed effects (Sweden as reference) and are weighted to reflect population distribution. Models compare individuals with undiagnosed diabetes (uDM) to: (1–2) normoglycemic individuals, (3–4) individuals with prediabetes, (5–6) individuals with diagnosed diabetes (dDM). Columns (1), (3), and (5) adjust for socio-demographic characteristics; Columns (2), (4), and (6) additionally control for health behaviors, health conditions, and healthcare usage. | | | | | | | |

**Source:** World Bank (2023)

eFigure 1. Percentage of GDP Spent on Healthcare by Country: 2015 vs. 2022/2023

**Source:** OECD (2023), World Bank (2023)

eFigure 2. Number of General Practitioners (GPs) per 1,000 Inhabitants: 2015 vs. 2021/2022

**Source:** OECD (2023), World Bank (2023)

eFigure 3. Percentage of Out-of-Pocket (OOP) Payments by Country: 2015 vs. 2022/2023
